# Soluble alpha-enolase activates monocytes by CD14-dependent TLR4 signalling pathway and exhibits a dual function

**DOI:** 10.1038/srep23796

**Published:** 2016-03-30

**Authors:** Clément Guillou, Manuel Fréret, Emeline Fondard, Céline Derambure, Gilles Avenel, Marie-Laure Golinski, Mathieu Verdet, Olivier Boyer, Frédérique Caillot, Philippe Musette, Thierry Lequerré, Olivier Vittecoq

**Affiliations:** 1 INSERM, U905 & Normandy University, Institute for Research and Innovation in Biomedicine (IRIB), Rouen, France; 2 Rouen University Hospital, Department of Rheumatology, Rouen, France; 3 Rouen University Hospital, Department of Dermatology, Rouen, France; 4 Rouen University Hospital, Department of Immunology, Rouen, France

## Abstract

Rheumatoid arthritis (RA) is the most common form of chronic inflammatory rheumatism. Identifying auto-antigens targeted by RA auto-antibodies is of major interest. Alpha-enolase (ENO1) is considered to be a pivotal auto-antigen in early RA but its pathophysiologic role remains unknown. The main objective of this study was to investigate the *in vitro* effects of soluble ENO1 on peripheral blood mononuclear cells (PBMC) from healthy donors and RA patients in order to determine the potential pathogenic role of ENO1. ELISA, transcriptomic analysis, experiments of receptor inhibition and flow cytometry analysis were performed to determine the effect, the target cell population and the receptor of ENO1. We showed that ENO1 has the ability to induce early production of pro-inflammatory cytokines and chemokines with delayed production of IL-10 and to activate the innate immune system. We demonstrated that ENO1 binds mainly to monocytes and activates the CD14-dependent TLR4 pathway both in healthy subjects and in RA patients. Our results establish for the first time that ENO1 is able to activate *in vitro* the CD14-dependent TLR4 pathway on monocytes involving a dual mechanism firstly pro-inflammatory and secondly anti-inflammatory. These results contribute to elucidating the role of this auto-antigen in the pathophysiologic mechanisms of RA.

Rheumatoid arthritis (RA) is a chronic inflammatory disorder characterized by chronic inflammation and synovial hyperplasia, leading to destruction of the cartilage and bone. Considered as an autoimmune disease, various auto-antigens have been shown to be the target of synovial T cells and auto-antibodies are present in patient’s serum and synovial fluid. Several auto-antibodies have been detected during RA development^1^ such as rheumatoid factors (RF) in the serum and synovial fluid of RA patients^2^ with good sensitivity (75–90%) but poor specificity (40%). Highly specific auto-antibodies (98%) known as anti-citrullinated protein/peptide auto-antibodies (ACPA) have been identified^3^. ACPA are detected in early RA, sometimes 10 years before the first clinical features^4^. Citrulline residues are produced through a post-translational modification of arginines catalysed by peptidyl-arginine deiminases (PAD). This modification changes the antigenicity of the protein. Several citrullinated auto-antigens, including fibrinogen, vimentin and alpha-enolase (ENO1)^5^, have been reported as target antigens of ACPA in the synovial tissue of RA patients. More recently, we and others have shown that the auto-antibody repertoire in early untreated RA is directed against several categories of antigens, notably some enzymes of the glycolytic pathway such as aldolase, phosphoglycerate-kinase 1, glucose-6-phosphate isomerase (G6PI) and ENO1, and chaperone molecules including calreticulin, HSP-60 and BiP^6^. The pattern of reactivity is variable among this panel of auto-antigens since they may be recognised either in their citrullinated or their native form, while some of them, such as BiP and ENO1, are the target of auto-antibodies that recognise both their citrullinated and native forms.

ENO1 is a major auto-antigen. Indeed, auto-antibodies against ENO1 are found in sera from patients with early RA^7^. Complementary studies have shown that citrullination of ENO1 is crucial for its auto-antigenicity since 46% of tested RA sera showed reactivity against the citrullinated form while reactivity against the non-citrullinated form ranged from 13 to 22%^7^,^8^.

ENO1 is a highly conserved glycolytic enzyme. It is a multifunctional protein that also plays the role of plasminogen receptor when expressed on the cell surface, which suggests that it might exhibit an important role in the modulation of the fibrinolytic system in RA pathophysiology^9^. Myc binding protein 1 (MBP-1), an alternative translation of ENO1 RNA, may be found in the nucleus, where it acts as a transcriptional repressor of the c-myc proto-oncogene, with subsequent regulation of cell growth and differentiation^10^.

The involvement of some auto-antigens in RA pathophysiology has been investigated. Indeed, fibrinogen, another major auto-antigen in RA, induces a pro-inflammatory response since the molecule has the ability to enhance pro-inflammatory cytokine production by PBMC^11^. In the DR4-IE mice model, citrullinated fibrinogen was demonstrated to be arthritogenic^12^, which was not observed with the unmodified protein. Moreover, G6PI was also demonstrated to be pro-inflammatory in a normal mouse model^13^. Furthermore, histone 2B (H2B), recently discovered as a new auto-antigen in RA, also has pro-inflammatory properties. Indeed, H2B citrullination increases innate immunostimulatory capacity and immune complexes containing citrullinated histones both activate macrophage cytokine production and propagate NETosis. Interestingly, in contrast to previous auto-antigens, BiP was characterised *in vitro* with immunomodulatory properties on PBMC from healthy donors^14^ and *in vivo* in the collagen induced arthritis mouse model^15^.

All those data suggest that most auto-antigens display pro-inflammatory effects and/or are arthritogenic. These effects are more often observed when the protein is citrullinated. But the pattern of reactivity appears to be variable according to the auto-antigen and its citrullinated or uncitrullinated form. Concerning ENO1, in the context of a specific HLA background, as observed in the DR4-IE transgenic mouse model, both forms of the auto-antigen were demonstrated to be arthritogenic^16^. However, to our knowledge, no data are available on the effect of ENO1 immune system cells from healthy donors and RA patients.

To investigate this issue, we analysed the *in vitro* effects of ENO1 on PBMC from healthy donors and RA patients. We showed that ENO1 triggers firstly inflammation, through the production of pro-inflammatory cytokines and chemokines, and then a delayed and extended anti-inflammatory effect, with IL-10 production. These effects are finally mediated by activation of the CD14-dependent TLR4 pathway on monocytes which play a key role in the pathophysiology of RA.

## Results

### ENO1 induces early TNF-α and delayed IL-10 production in PBMC from healthy donors and RA patients

**Figure 1:**
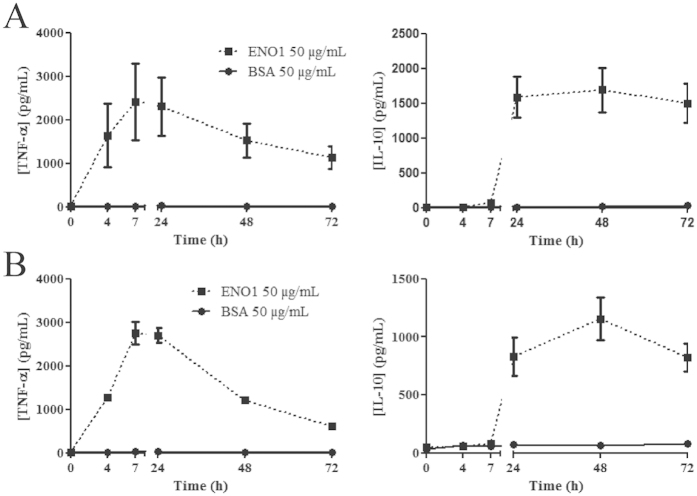
ENO1 induces early TNF-α and delayed IL-10 production in PBMC from healthy donors and RA patients. To investigate a potential pro- or anti-inflammatory effect of ENO1, PBMC from healthy donors (**A**) or RA patients (**B**) were incubated with ENO1 (50 μg/mL) or BSA (50 μg/mL) and cultured for 72 hours. Supernatants were removed at different times (H0, H4, H7, H24, H48, and H72) and TNF-α and IL-10 levels were measured by ELISA. Data are representative of three experiments for healthy donors and RA patients. At least ten experiments were reproduced for healthy donors. Data are expressed as mean ± SEM (n = 3).

To investigate a potential pro- and/or anti-inflammatory effect of ENO1, PBMC from healthy donors or RA patients were cultured with ENO1 (according to the dose-response experiment, the most appropriate dose of ENO1 to be investigate in next experiments was 50 μg/ml (Sup. Fig. 1)) or control BSA (50 μg/mL). Using ELISA, we assayed the levels of TNF-α and IL-10 produced at different time points in culture supernatants. In PBMC from healthy donors (Fig. 1A) and RA patients (Fig. 1B), ENO1 induced early TNF-α production which peaked at H7 followed by IL-10 production at H24, compared to control BSA. The same cytokine profile was observed with citrullinated ENO1 (Sup. Fig. 2). Since we observed the same cytokine pattern with both forms of ENO1, we decided to perform the next experiments exclusively with the native form of ENO1. Indeed, the degree of ENO1 citrullination may be variable even though we carried out the same *in vitro* procedure. It can be excluded that the observed effect was due to residual LPS that might remain after ENO1 production in *E.coli* because this effect on ENO1 stimulated PBMC was not inhibited by the LPS inhibitor polymixin B (250 μg/mL), which was the case for LPS-stimulated PBMC (1 μg/mL) (Sup. Fig. 3). Moreover, to confirm the absence of residual LPS effect in ENO1 solution, we showed that ENO1b (produced in LPS-free baculovirus-insect cell expression system) had a similar effect to ENO1 on cytokine production by PBMC, whereas PAG 1- (pregnancy-associated glycoprotein 1 precursor, produced in *E.coli*) stimulated PBMC did not produce TNF-α or IL-10 (Sup. Fig. 4). Hence, according to these experiments, both forms (native and citrullinated) of ENO1 appear to induce in PBMC an early pro-inflammatory response followed by a delayed and sustained anti-inflammatory response.

### ENO1 firstly involves inflammation, innate immune response and TLR pathways

**Figure 2:**
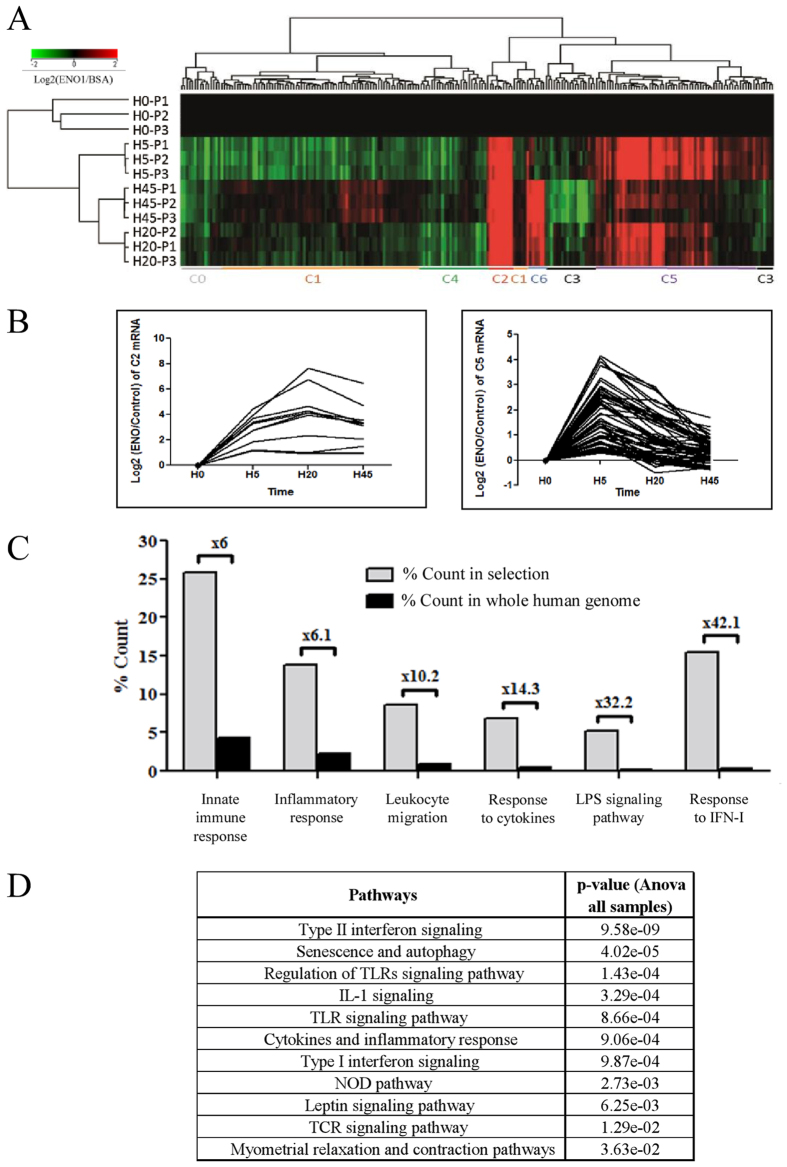
Identification of differential gene expression profiling relative to time in ENO1 treated PBMC compared to BSA incubated PBMC. PBMC from three healthy donors (P1-P3) were treated by ENO1 (50 μg/mL) or control BSA (50 μg/mL) according to time (H0 ,H5, H20, H45). RNA was extracted, labelled by Cy-3 (control BSA samples) or Cy-5 (ENO1 samples) and co-hybridised on 4 × 44 K whole human genome microarray (Agilent technologies) (**A**) 223 mRNA whose dysregulation was significantly associated with time (ANOVA with FDR correction; p-value < 0.05) were represented by Hierarchical Clustering (Pearson coefficient metric and Wards linkage). K-means (Pearson coefficient metric) allowed us to identify seven transcript clusters according to their expression profiling: C0-C6 identified by coloured lines. (**B**) Gene expression profiling of C2 (left) and C5 (right) transcript clusters according to time. (**C**) Pie chart of Gene Ontology analysis from C2 (n = 12) and C5 (n = 60) transcript subsets. GO terms are significantly enriched according to standard hypergeometric distribution (p-value < 0.05). (**D**) Curated-WikiPathways analysis from C2 (n = 12) and C5 (n = 60) transcript subsets. WikiPathways are significantly enriched according to standard hypergeometric distribution (p-value < 0.05).

To investigate the mechanisms involved in ENO1 stimulated PBMC, we compared the transcriptome of ENO1 cultured PBMC with the transcriptome of BSA cultured PBMC in a time manner (H0, H5, H20, H45). This analysis revealed 223 genes significantly dysregulated in PBMC stimulated by ENO1, compared to PBMC incubated with BSA, in a time manner (Fig. 2A, Sup. Table 1). These genes were classified into seven different statistically relevant clusters depending on their expression over time. Among them, only two clusters (C2 and C5), consisting of 72 up-regulated genes (Fig. 2B), allowed us to identify various significant biological processes and pathways involved during PBMC stimulation by ENO1 (Fig. 2C,D). Among these six highly relevant biological processes identified by the gene ontology (GO) enrichment analysis performed for clusters 2 and 5 we found: activation of the innate immune system (6-fold enrichment compared to whole human genome, 15 related genes), inflammatory response (6.1-fold enrichment, 8 related genes), leukocyte chemotaxis and migration (10.2-fold enrichment, 5 related genes) and response to cytokines (14.3-fold enrichment, 14 related genes). Interestingly, we found the process of response to LPS (32.2-fold enrichment, 3 related genes) and response to IFN-I (42.1-fold enrichment, 9 related genes) (Fig. 2C, Sup. Table 2). Furthermore, biological processes can be correlated with statistically relevant molecular pathways. The involvement of pathways such as IFN-II signalling, regulation of TLR signalling pathway, IL-1 signalling, cytokine and inflammatory response and IFN-I signalling suggest that ENO1 has a pro-inflammatory effect on innate immune system cells (Fig. 2D), especially monocytes.

### ENO1 binds to monocytes and exhibits an early pro-inflammatory effect and a delayed anti-inflammatory effect

**Figure 3:**
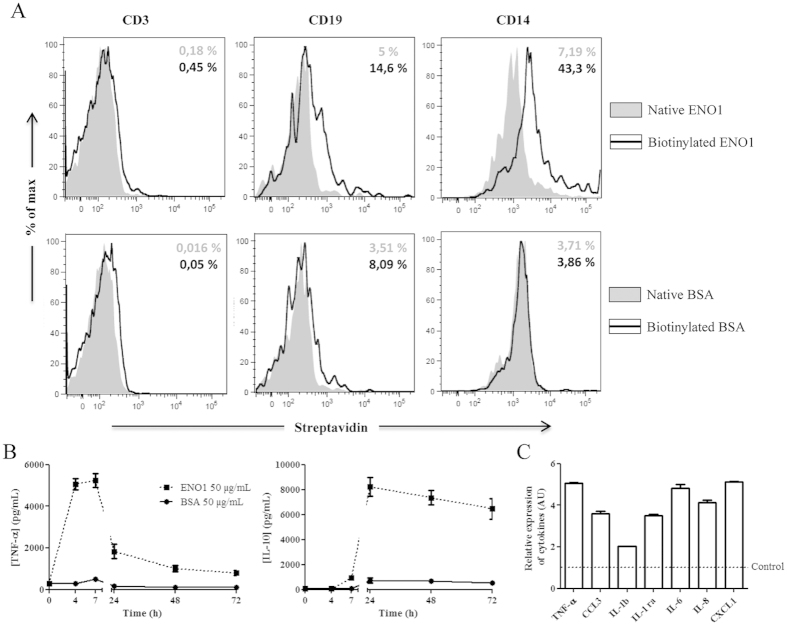
ENO1 binds to monocytes and exhibits a pro- and anti-inflammatory effect. To identify cellular targets of ENO1, native and biotinylated ENO1 (50 μg/mL) or native and biotinylated BSA (50 μg/mL) were cultured with PBMC from healthy donors for 24 hours. Filled plot corresponds to native proteins and black line corresponds to biotinylated proteins. Expression levels of streptavidin on cell surface were determined by flow cytometry on T cells, B cells and monocytes, using CD3, CD19 and CD14 labelling respectively. One representative result of three independent experiments is presented. The percentage in grey corresponds to streptavidin positive cells of native protein experiments. The percentage in black corresponds to streptavidin positive cells of biotinylated proteins experiments (**A**). After negative selection of PBMC from healthy donors, 1.10^6^ monocytes were cultured with ENO1 (50 μg/mL) or BSA (50μg/mL). Supernatants were removed at different times (H0, H4, H7, H24, H48, and H72) and TNF-α and IL-10 production was measured by ELISA. Data are expressed as mean ± SEM (n = 3) (**B**). Monocytes were stimulated with ENO1 (50 μg/mL) or control BSA (50 μg/mL) for seven hours. Supernatants were removed and the level of 36 cytokines and chemokines was measured using proteome profiler approach. The histogram represents rates of cytokines (TNF-α, IL-1β and IL-6) and chemokines (CCL3, IL-8, CXCL1) which are at least 2-fold that of the BSA control (dotted line). Data are expressed as mean ± SEM (n = 2) (**C**).

To identify the cellular target of ENO1 in PBMC, we incubated biotinylated ENO1 or biotinylated BSA with PBMC from healthy donors. Biotinylation of both proteins was checked by Western blot analysis (data not shown). We showed that ENO1 did not bind to T cells but only to a small percentage of B cells, whereas its binding to monocytes was particularly relevant (Fig. 3A). Moreover, the cytokine profile obtained with monocytes cultured with ENO1 was similar to that observed with PBMC. But TNF-α and IL-10 levels were respectively 2- and 5-fold higher than those measured with PBMC (Fig. 3B). Furthermore, using a proteomic approach, we showed that ENO1-stimulated monocytes produced a large panel of pro-inflammatory cytokines and chemokines. Indeed, compared to monocytes cultured with BSA, ENO1-incubated monocytes released 2- to 5-fold higher levels of pro-inflammatory cytokines such as IL-1β, IL-6 in addition to TNF-α (Fig. 3C), which is in accordance with results obtained by transcriptomic approach. These ENO1 treated monocytes also produced 4- to 5-fold higher levels of chemokines, especially those having a pivotal role in leukocyte migration to inflammatory sites, such as CCL3, CXCL1 and IL-8 (Fig. 3C). Moreover, IL-1Ra levels, which have anti-inflammatory properties, were 3-fold higher in monocytes treated by ENO1 compared to those incubated with BSA. These results suggest that ENO1 mainly binds to monocytes, sufficient to produce an initial inflammatory response. In this respect, ENO1 had no effect on T cells, B cells or monocytes-depleted PBMC since no cytokine was produced *in vitro* (Sup. Fig. 5). According to transcriptome analysis, the involvement of biological processes such as regulation of TLR signalling pathway as well as LPS response might suggest a TLR4-dependent inflammatory response.

### ENO1 and LPS have a similar effect on TNF-α and IL-10 secretions

**Figure 4:**
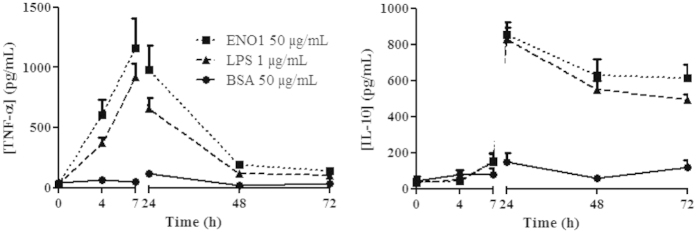
ENO1 and LPS have similar cytokine profiles. To evaluate the different cytokine profiles of ENO1 and LPS, PBMC from healthy donors were stimulated with ENO1 (50 μg/mL), LPS (1 μg/mL) or BSA (50 μg/mL). Supernatants were removed at different times (H0, H4, H7, H24, H48, and H72) and TNF-α and IL-10 levels were measured by ELISA. Data are expressed as mean ± SEM (n = 3).

Our data suggests that ENO1 might have a TLR-mediated inflammatory effect, more specifically by TLR4, which is known to bind LPS. We hypothesised a potential similar cytokine secretion between PBMC stimulated by ENO1 and LPS. With this regard, we cultured PBMC from healthy donors with ENO1 (50 μg/mL) or LPS (1 μg/mL, dose corresponding to similar cytokine production observed with ENO1 (Sup. Fig. 6)) or control BSA (50 μg/mL). The level of cytokine production was assessed in culture supernatants at different times by ELISA. Interestingly, ENO1 and LPS induced similar cytokinic profiles with early production of TNF-α and extended release of IL-10, while BSA had no effect, as expected (Fig. 4). These data suggest that ENO1 has an LPS-like effect with a dual function including an early pro-inflammatory effect and a late anti-inflammatory effect.

### *In vitro* effect of ENO1 is TLR4 pathway-dependent

**Figure 5:**
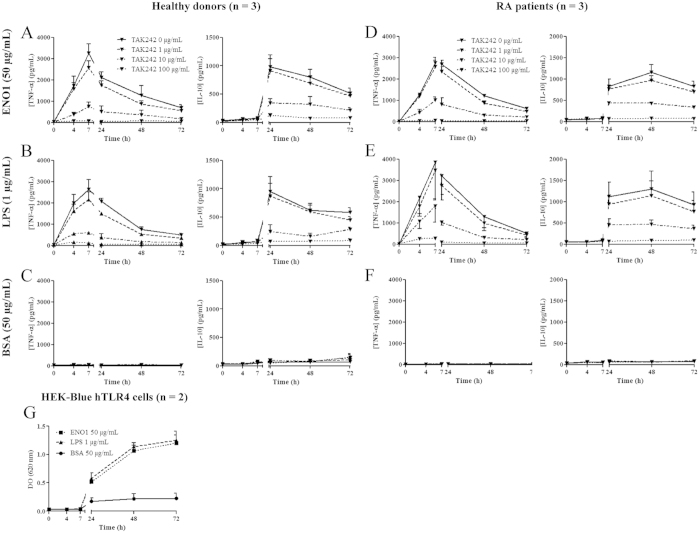
Effects induced by ENO1 are TLR4 pathway-dependent. 1.10^6^ PBMC from healthy donors (**A–C**) and RA patients (**D–F**) were cultured with ENO1 (50 μg/mL), LPS (1 μg/mL) or BSA (50 μg/mL) with or without increasing doses of TAK242 (0, 1, 10 and 100 μg/mL). Supernatants were removed at 0–72 hours and cytokine (TNF-α and IL-10) levels were measured by ELISA. Data are expressed as mean ± SEM (n = 3). 1.10^6^ of HEK-Blue hTLR4 cells were cultured with ENO1 (50 μg/mL), LPS (1 μg/mL) or BSA (50 μg/mL). Supernatants were removed (200 μL) at 0–72 hours, and TLR4 activation was quantified by QUANTI-Blue. Data are expressed as mean ± SEM (n = 2) (G).

Our results revealed that ENO1 induces pro-inflammatory and anti-inflammatory cytokine secretions. Transcriptomic approach showed involvement of TLR-dependent pathways combined with LPS response. Moreover, there was a similar cytokine profile in PBMC induced by ENO1 and LPS. Taken together, these data suggest that the cellular response to ENO1 could be mediated by the TLR4 pathway. To confirm this hypothesis, PBMC from healthy donors (Fig. 5A–C) or RA patients (Fig. 5D,E) were cultured with ENO1 (50 μg/mL), LPS (1 μg/mL) or control BSA (50 μg/mL) with different doses (0, 1, 10 or 100 μg/mL) of TAK242, a TLR4 inhibitor. Cytokine production was assessed in culture supernatants at different times by ELISA. We showed that TAK242 inhibits the production of TNF-α and IL-10 in PBMC cultured with ENO1 and LPS in a dose dependent manner. Indeed, incubation with increasing concentration of TAK242 partially or totally inhibited TNF-α and IL-10 production as observed for 10 μg/mL and 100 μg/mL respectively, whereas 1 μg/mL had no effect (Fig. 5). These results suggest that ENO1 binds TLR4 to induce a pro-inflammatory response. To confirm the ENO1-TLR4 pathway specificity, we performed inhibitory experiments on TLR1 and TLR5 pathways. PBMC stimulated by ENO1 (50 μg/mL), LPS (1 μg/mL) or BSA (50 μg/mL) and incubated with TLR1, TLR2 or TLR5 neutralizing antibodies (5 μg/mL) exhibited the same cytokine profiles as those observed without blocking antibodies (Sup. Figs 7–9). Furthermore, these data were confirmed using this time a cell-line with exclusive expression of TLR4, referred to as HEK-Blue hTLR4 cells (Fig. 5G). In order to compare the binding of ENO1 and LPS to TLR4, we performed a competitive binding assay with ENO1 and FITC-labelled LPS using HEK-Blue hTLR4 cells. We showed that ENO1 does not impact the binding of FITC-labelled LPS to TLR4 but competes with the APC-labelled anti-TLR4 antibody (Sup. Fig. 10). These results suggest that ENO1 binds TLR4 but not in the same manner as LPS.

### ENO1 induces the CD14-dependent TLR4 pathway

**Figure 6:**
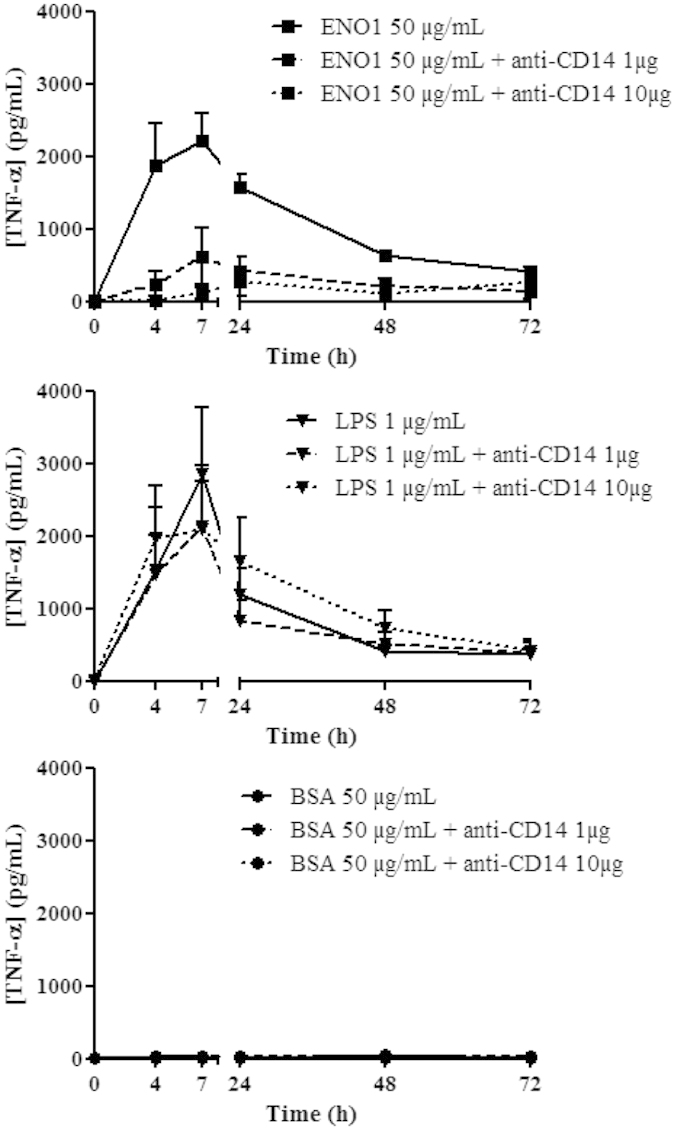
ENO1 induces the CD14-dependent TLR4 pathway. 1.10^6^ PBMC from healthy donors were cultured with ENO1 (50 μg/mL), LPS (1 μg/mL) or BSA (50 μg/mL) with different doses of anti-CD14 blocking antibody (0, 1 or 10 μg). Supernatants were removed at different times (H0, H4, H7, H24, H48, and H72) and TNF-α and IL-10 production was measured by ELISA. Data are expressed as mean ± SEM (n = 3).

We showed in this work that ENO1 binds to monocytes *via* TLR4. It was previously shown that two different TLR4 pathways exist and that one is CD14-dependent^17^. LPS can bind TLR4 using these two pathways. To determine whether ENO1 induced both pathways or only one of them, PBMC were incubated with ENO1 (50 μg/mL), LPS (1 μg/mL) or control BSA (50 μg/mL) and with increasing doses (0, 1 or 10 μg/mL) of anti-CD14 antibodies. Using ELISA, we evaluated at different times the level of TNF-α in stimulated PBMC supernatants. While blockade of CD14 does not modify TNF-α production in LPS-stimulated PBMC, it inhibits in a dose dependent manner TNF-α production in PBMC cultured with ENO1 (Fig. 6). These data demonstrate that the *in vitro* effect of ENO1 is mediated by the CD14-dependent TLR4 pathway.

## Discussion

This study demonstrates for the first time that ENO1 has pro- and anti-inflammatory effects on PBMC and notably on monocytes from healthy donors and RA patients. These effects are mediated through TLR4 pathways, leading to the conclusion that ENO1 is a new ligand of TLR4.

We and others have demonstrated that the auto-antibody repertoire in early untreated RA is directed against several antigens, notably ENO1^7^. Auto-antibodies against native ENO1 are present in sera from patients with early RA^7^ but also in a large variety of infectious and autoimmune diseases^18^. While the role of auto-antibodies against citrullinated ENO1 is better understood in RA, that of auto-antibodies recognising the native form of ENO1 remains unclear^8^. It has been shown that the target of these auto-antibodies can be found in human serum^19^. Some of its functions, such as the role of plasminogen receptor and its glycolytic activity, can be implicated in RA pathophysiology. Indeed, a study has demonstrated a decrease in both glycolytic activity and fibrinolytic activity in RA patients^20^. To date, no studies have investigated the potential effects of ENO1 in cells from the immune system. In the present study, we investigated the impact of ENO1 on the cytokine production of PBMC from healthy donors and RA patients. We showed that ENO1 induces early production of pro-inflammatory cytokines such as TNF-α (Fig. 1) and IL-1β (data not shown) and also delayed production of anti-inflammatory cytokines such as IL-10 (Fig. 1) and IL-1Ra (data not shown). To confirm the possible pro- or anti-inflammatory role of ENO1, we performed a transcriptomic analysis of PBMC incubated with ENO1. This approach revealed that ENO1 effects highly involve several pathways of inflammation and we focused on TLR pathway regulation and response to LPS pathway (Fig. 2). In view of these results, to identify the cell population targeted by ENO1, we performed a cytometry analysis which showed that ENO1 binds mainly to monocytes (Fig. 3A). Thereafter, monocytes alone are able to produce a panel of pro-inflammatory cytokines such as TNF-α, IL-1β and IL-6 in response to ENO1 (Fig. 3B,C). These cytokines have a pivotal role in RA pathophysiology including perpetuation of inflammation^21^,^22^,^23^. ENO1 stimulated monocytes also produce multiple chemokines such as CCL3, IL-8 and CXCL1 (Fig. 3C) which are involved in the acute inflammatory state, in the recruitment and activation of leukocytes^24^,^25^. Otherwise, we observed higher IL-1Ra production, known to be anti-inflammatory, in the supernatants of monocytes cultured with ENO1, suggesting a dual role of ENO1 *in vitro*. However, a 50% inhibition of these IL-1β induced biological responses requires amounts of IL-1Ra up to 100-fold in excess of the amounts of IL-1β^26^. Taken together, these results suggest that ENO1 exhibits a significant pro-inflammatory, followed by an anti-inflammatory action on monocytes likely to be close to that of LPS. We confirmed this hypothesis by the strictly similar profile of TNF-α and IL-10 production by PBMC stimulated with LPS or ENO1 (Fig. 4). Cytokine production was not due to residual LPS in the buffer of ENO1 (produced in *E. coli*.) since we showed similar results with or without polymixine B, an LPS inhibitor (Sup. Fig. 3). Moreover, the same cytokine profile was observed using ENO1 produced with LPS-free baculovirus-insect cell expression system and PAG 1- (produced in *E.coli*) stimulated PBMC did not produce cytokines (Sup. Fig. 4). In addition, transcriptomic analysis suggested the involvement of TLR pathways. In this respect, TLR4 is known to bind LPS and to be expressed almost exclusively in monocytes^27^. These well-documented data led us to evaluate the binding of ENO1 to TLR4. We have provided herein several arguments confirming such binding, since TLR4 blocking experiments as well as ENO1 effects on a specific cell line exclusively harbouring TLR4 were in favour of an action mediated through TLR4 (Fig. 5). TLR4 is able to bind misfolded or unfolded proteins and to activate the appropriate pathways. The ENO1 conformation obtained after production in *E. coli* might activate the TLR4 signalling pathway, independently of a specific role of this protein. Our experiments with ENO1 produced with baculovirus-insect cell expression system have shown similar results with production of TNF-α and IL-10 (Sup. Fig. 4). Moreover, PBMC stimulated with PAG 1, produced in *E. coli* with a similar molecular weight as ENO1, did not produce TNF-α or IL-10, in a comparable manner to BSA (Sup. Fig. 4). In this regard, ENO1 seems to have the same mechanisms of action as LPS *in vitro*. Effects of LPS are mediated through two pathways for TLR4 signalling, *i.e.* MyD88 dependent pathway where LPS binds directly the TLR4-MD2 complex and the MyD88 independent pathway which requires CD14. It has been suggested that activation of the CD14-dependent TLR4 pathway can induce production of IFN-β, contrary to the CD14-independent TLR4 pathway^17^. CD14 inhibition experiments allowed us to conclude that ENO1, unlike LPS, only activates the CD14-dependent TLR4 pathway of monocytes to induce an important inflammatory response in healthy subjects and in RA patients. We were not able to show production of IFN-β by ENO1 stimulated cells using ELISA (data not shown), but transcriptomic analysis showed a 42.1-fold enrichment of the response to IFN-I process, IFN-β forming part of type I IFNs. Finally, both forms (native and citrullinated) of ENO1 seem to have the same effects on the cytokine production of PBMC (Sup. Fig. 2). This observation suggests that citrullination does not alter ENO1 binding to TLR4 or subsequent inflammatory response, while a loss of function has been reported for other proteins such as MBP-1 in multiple sclerosis^28^. Further to our present results that show ENO1 binding to TLR4, it would be interesting to study the direct interaction of ENO1 TLR4 and CD14 by surface plasmon resonance technology for better understanding of ENO1 action and to highlight the specific pathways involved.

The initial pro-inflammatory properties of ENO1 have been suggested in previous reports. Indeed, it has been shown that the up-regulated surface expression of ENO1 in haematopoietic cells play an important role in the inflammatory process by the rapidly up-regulated cell-surface expression of ENO1 on human monocytes by rapid translocation of ENO1 to the cell surface from cytosol after stimulation by LPS^29^. Bae *et al.* also showed that cell-surface expression of ENO1 has an increased number of monocytes and macrophages isolated from RA patients and that antibodies against ENO1 can stimulate these cells to produce higher amounts of pro-inflammatory mediators such as TNF-α, IL-1α/β, IFN-γ, and PGE2^30^.

Given previous data from the literature and ours, ENO1 seems to exert a pro-inflammatory effect through two mechanisms. The first is represented by cell-surface ENO1-upregulation under inflammatory *stimuli* such as LPS or pro-inflammatory cytokines. Monocytes and macrophages are the cells that preferentially express ENO1 in PBMC and synovial fluid mononuclear cells derived from RA patients^30^. At this level, ENO1 acts as a plasminogen receptor, which results in enhanced plasmin proteolytic activity at the cell surface. This ENO1-mediated increase in plasmin formation promotes extra-cellular matrix degradation and migration of monocytic cells. Such a mechanism facilitates the migration of monocytes into the targeted tissue, which has been shown in inflamed lung^29^, but not yet in inflamed synovial tissue. Interestingly, other auto-antigens, namely annexin II and H2B, have been identified as binding sites for plasminogen^31^. Like ENO1, H2B also play a role during monocyte/macrophage recruitment^32^.

The second mechanism is highlighted in the present study. Once again, it has been shown that other major auto-antigens in RA, fibrinogen and H2B, also induced an early inflammatory response through binding of monocytes to TLR4^33^,^34^. Moreover, it has been demonstrated that auto-antibodies against fibrinogen can form immune complexes which not only induce robust cytokine production by macrophages as mentioned previously, but could also facilitate enhanced TNF-α production by co stimulating TLR4 and FcγR^35^,^36^. Thus, like fibrinogen and H2B, the ENO1 auto-antigen is a new ligand of TLR4. Our results concerning the new role of soluble ENO1 open interesting perspectives and it seems relevant, as for fibrinogen, to study the role of ENO1/anti-ENO1 antibody immune complexes in RA pathophysiology.

ENO1 appears to have an initial pro-inflammatory effect after binding to TLR4 and activation of its CD14-dependent pathway. However, we have also shown that production of IL-10 by cells stimulated by LPS and ENO1 is late and extended. This effect appears particularly interesting and highlights the potential dual role of ENO1, as LPS. After their pro-inflammatory effect, ENO1 and LPS could have an immunomodulatory action. Indeed, Wang *et al.* showed in 2015 that LPS had protective effects against the development of diabetes in NOD diabetic mice^37^. These effects involved regulatory T lymphocyte (Treg) induction, down-regulation of TLR4 and the potential emergence of a tolerogenic dendritic cell (DC) subset. Moreover, Pace *et al.* demonstrated in 2015 that LPS was significantly able to induce Foxp3 expression in lymphocytes and in total CD4^+^CD25^−^ lymphocytes from asthmatic patients and controls^38^. ENO1 could therefore have immunomodulatory effects *via* its dual role and these mechanisms. In this context, in 2015 we showed that prophylactic injection of ENO1 in a collagen-induced arthritis (CIA) mouse model allowed significant prevention of arthritis severity^39^.

In summary, we have shown for the first time that ENO1 has a dual effect on monocytes by activating the CD14-dependent TLR4 pathway. In this regard, ENO1 might have a pivotal role at different stages of RA pathophysiology. Indeed, ENO1 seems to play a role in the initial phase of RA, especially by activation of the innate immune system. Concerning the chronic phase of the disease, both forms (native and citrullinated) of ENO1 as well as auto-antibodies recognizing citrullinated ENO1 might exert pro-inflammatory action since ENO1 promotes leukocyte recruitment and triggers pro-inflammatory cytokine production. Moreover, ACPA-containing immune complexes are able to trigger TNF-α secretion through engagement of FcγR IIa receptors in macrophages^35^, which are essential for the perpetuation of inflammation. Finally, ENO1 could even have a role in other inflammatory diseases such as inflammatory bowel diseases or cancer in which anti-ENO1 antibodies have been detected^40^,^41^. Thus, ENO1 might be one of the actors that corroborate the self-maintaining inflammatory hypothesis in RA. Nevertheless, the dual effect of ENO1 and notably its delayed anti-inflammatory action might have an important therapeutic value in the prevention and treatment of RA through the emergence of regulatory cell populations in this pathology, which remains to be investigated.

## Methods

### Patients

PBMC were provided from buffy coats from the French Blood Agency for healthy blood donors (n = 3) and from whole venous blood for RA patients (n = 3). RA patients were recruited to the VErA (Very Early rheumatoid Arthritis) cohort and followed for at least 10 years. All experimental protocols were approved by the Upper Normandy Ethics Committee (file 95/138/HP). All patients fulfilled the 2010 ACR/EULAR criteria^42^ and gave their informed consent. Methods were carried out in accordance with the approved guidelines.

### ENO1

Human recombinant ENO1 production in *E. coli* was performed by Delphi Genetics (Gosselies, Belgium). This ENO1 was suspended in PBS 1X with 1.3 M of urea for solubilisation (final concentration: 1 mg/mL) and endotoxin was removed by chromatic resin by the manufacturer. Using a commercial kit (Qcl chromogenic LAL kit, Lonza), the titre of LPS was <1 EU/mL. To check some experiments, human recombinant ENO1 was also produced with LPS-free baculovirus-insect cell expression system (ENO1b) by ProteoGenix (Schiltigheim, France). This ENO1b was suspended in PBS 1X.

### PAG 1

*Bos Taurus* recombinant PAG 1 production in *E. coli* was performed by ProteoGenix (Schiltigheim, France). This PAG 1 was suspended in PBS 1X with 2 M of urea for solubilisation (final concentration: 1 mg/mL). We removed endotoxin by EndoTrap red Endotoxin Removal Kit (Hyglos). Using a commercial kit (Qcl chromogenic LAL kit, Lonza), the titre of LPS was <1 EU/mL.

### ENO1 citrullination

We incubated 30 μg of ENO1 with PAD (Sigma) at 7 U/mL and citrullination buffer (Tris 0.1 M, DTT 5 mM, CaCl2 10 mM) at 0.3 mg of protein/mL for 3 hours at 50 °C. To stop the reaction, we added 20 mM of EDTA.

### Controls

Two controls were used: Bovine Serum Albumin (BSA) and PAG 1 protein (pregnancy-associated glycoprotein 1 precursor, *Bos Taurus* sequence), produced in *E. coli* (Proteogenix, Schiltigheim, France).

### Purification of cells from PBMC

After extraction of PBMC, monocytes, T cells or B cells were purified by negative selection using human monocyte Dynal beads, human T cell Dynal beads or human B cells Dynal beads respectively, according to the manufacturer’s instructions (Invitrogen). The purity of monocytes, T cells and B cells was checked after isolation by flow cytometry using anti-CD14, anti-CD3 and anti-CD19 antibodies respectively. The purity of the cells was >85% as determined by immunostaining. Monocytes-depleted PBMC were obtained by removal of CD14^+^ cells among PBMC using EasySep Human CD14 Positive Selection Kit (Stemcell Technologies). Absence of monocytes (0.31%) was checked by flow cytometry using anti-CD14 antibody.

### Cell cultures

#### To investigate ENO1 effects on PBMC

PBMC from healthy donors and RA patients were isolated by density gradient centrifugation over Ficoll-Hypaque (Lymphoprep, Oslo, Norway). 1.10^6^ cells were cultured in 12-well plates with 1 mL of RPMI 1640 supplemented by 10% fetal calf serum, 1% glutamine and 0.5% antibiotic (penicillin and streptomycin). To determine the optimal dose, different concentrations of ENO1 were tested (5, 10, 50 and 100 μg/mL) on the PBMC of healthy donors. Afterwards, PBMC from healthy donors or RA patients were cultured with native or citrullinated ENO1 (50 μg/mL), or BSA (50 μg/mL) as control for 72 hours in a 5% CO_2_ incubator at 37 °C. Supernatants (200 μL) were removed at different times from 0 to 72 hours, aliquoted and frozen at −80 °C for further analysis by ELISA. To assess the hypothetical effect of residual LPS in ENO1 solution, PBMC from healthy donors were cultured with ENO1 (50 μg/mL) or LPS (1 μg/mL) and with or without polymixin B (250 μg/mL, dose sufficient to inhibit 10 μg/mL of LPS) for 72 hours. Supernatants were removed at different times and TNF-α level was measured by ELISA.

#### To assess ENO1 effect on monocytes, T cells and B cells

Monocytes, T cells and B cells were purified by negative selection using human monocyte, T cell and B cell Dynal beads respectively, according to the manufacturer’s instructions (Invitrogen). Monocytes, T cells and B cells from healthy donors (1.10^6^ cells/well) were cultured in 12-well culture plates with ENO1 (50 μg/mL) or control BSA (50 μg/mL) for 72 hours. Supernatants were removed (200 μL) at 0–72 hours, aliquoted and frozen at −80 °C for further analysis by ELISA and proteome profilers.

#### To compare ENO1 and LPS action

PBMC from healthy donors were cultured with ENO1 (50 μg/mL) or BSA (50 μg/mL) or LPS (1 μg/mL). Supernatants were removed (200 μL) at 0–72 hours, aliquoted and frozen at −80 °C for further analysis by ELISA.

#### To investigate the ENO1 mediated pathway

PBMC from healthy donors were incubated with TAK242 (TLR4 inhibitor) (Invivogen) at different doses (1, 10 and 100 μg/mL) for 1 hour, PAb-hTLR1 (TLR1 neutralizing antibody) (Invivogen) at 5 μg/mL, PAb-hTLR2 (TLR2 neutralizing antibody) (Invivogen), PAb-hTLR5 (TLR5 neutralizing antibody) (Invivogen) at 5 μg/mL or anti-CD14 antibody (Invivogen) at 1 or 10 μg/mL. Then, we added ENO1 (50 μg/mL), LPS (1 μg/mL) or control BSA (50 μg/mL) for 72 hours. Supernatants were removed (200 μL) at 0–72 hours, aliquoted and frozen at −80 °C for further analysis by ELISA.

#### HEK-Blue hTLR4 cells

HEK-Blue hTLR4 cells were cultured according to manufacturer’s instructions (Invivogen). These cells were obtained by co-transfection of the human TLR4, MD-2 and CD14 co-receptor genes, and an inducible SEAP (secreted embryonic alkaline phosphatase) reporter gene in HEK293 cells. Cells were cultured in DMEM high-glucose medium supplemented with 10% fetal bovine serum (FBS), 2 mM glutamine, 1X Normocin (InvivoGen), 1X HEK-Blue Selection (InvivoGen). 1.10^6^ cells were cultured with ENO1 (50 μg/mL), LPS (1 μg/mL) or BSA (50 μg/mL). Supernatants were removed (200 μL) at 0–72 hours, aliquoted and frozen at −80 °C for further analysis of TLR4 activation. Activation of TLR4 pathways was assessed by quantification of secreted SEAP with QUANTI-Blue^™^ (Invivogen) according to the manufacturer’s instructions.

### ELISA tests measuring TNF-α and IL-10 production

The supernatant levels of IL-10 and TNF-α were measured using human IL-10 DuoSet (R&D Systems) and human TNF DuoSet (R&D Systems) according to the manufacturer’s instructions.

### Proteome profiler

Supernatants removed from cultures of monocytes at 7 hours were used to assess the production of a panel of pro- and anti-inflammatory cytokines as well as of chemokines using a proteome profiler human cytokine array kit, panel A, according to the manufacturer’s instructions (R&D Systems).

### Biotinylation of ENO1

We used a biotinylation kit (EZ-Link® Micro Sulfo-NHS-Biotintylation Kit, Thermo Scientific), according to the manufacturer’s instructions to biotinylate ENO1 and BSA control.

### Flow cytometric analysis

#### To identify the cellular target of ENO1

To determine which cell types can bind ENO1, PBMC (1.10^6^ cells/mL) from healthy donors were cultured with biotinylated or native ENO1 (50 μg/mL) as well as with biotinylated or native BSA (50 μg/mL) for 24 hours. Then, cells were removed and washed with phosphate buffer saline (PBS). A fraction of cells was examined by direct double-staining with mAb against cell surface markers. Cells were incubated with saturating concentrations of Streptavidin-FITC and anti-CD3-PE corresponding to T cells, Streptavidin-FITC and anti-CD14-PE corresponding to monocytes, and Streptavidin-FITC and anti-CD19-PE corresponding to B cells for 30 minutes at 4 °C. Antibodies were obtained from PharMingen (San Diego, CA). After two washes with PBS, cells were suspended in PBS (500 μL). Analysis of cell surface marker expression was performed using a flow cytometer (Canto).

#### To study ENO1-TLR4 binding (competitive binding assay)

HEK-Blue hTLR4 cells were cultured in DMEM high-glucose medium supplemented with 10% fetal bovine serum (FBS), 2 mM glutamine, 1X Normocin^™^ (InvivoGen), and 1X HEK-Blue^™^ Selection (InvivoGen). 1.10^6^ cells were cultured 20 minutes at 4 °C with sucrose (1 mg) and different concentrations of ENO1 (25 μg, 500 μg or 2 mg). 55 μg of FITC-labelled LPS (Sigma Aldrich) and 10 μl of APC-labeled anti-TLR4 antibody (AbD Serotec) were added for 20 minutes at 4 °C. After two washes with PBS, cells were suspended in PBS (500 μL). Analysis of FITC and APC level was performed using a flow cytometer (Canto).

### mRNA isolation

PBMC from healthy donors (n = 3) were cultured with ENO1 (50 μg/mL) or control BSA (50 μg/mL). Cells were removed at different times (H0, H5, H20, H45) to carry out mRNA extraction. mRNA samples, referred to as ENO1 sample and BSA sample, were obtained using RNAqueous 4-PCR kit according to the manufacturer’s instructions (Ambion®, Austin, USA). The purity and integrity of RNA samples was assessed using a Bioanalyzer 2100 (Agilent Technologies).

### Gene expression analysis

Whole human DNA microarrays were used to analyse two-coloured gene expression profiling (4 × 44 k Whole Human Genome G4112F, Agilent Technologies). Each ENO1 sample was labelled by Cyanin 5 and co-hybridised with a Cyanin 3 labelled RNA control BSA according to the manufacturer’s instructions (Low Input QuickAmp Labeling Kit, Agilent Technologies). Briefly, 100 ng of total RNAs were labeled with cyanin-5 CTP (ENO1 sample) or cyanin-3 CTP (control BSA sample). Co-hybridization was performed at 65 °C for 17 hours using a hybridisation kit (Agilent Technologies). After wash steps, the microarrays were scanned with a 5 μM pixel size using the DNA Microarray Scanner GB (Agilent Technologies). Image analysis and the extraction of raw and corrected signal intensities (Lowess normalisation) were performed with Feature Extraction Software 10.5.1.1 (Agilent Technologies). Data were in agreement with the Minimum Information About a Microarray Experiment guidelines and were deposited in the Gene Expression Omnibus of the National Center for Biotechnology Information. Data are accessible using the following accession number: GSE68089.

### Data mining and functional analysis

Non-uniform spots and spots with saturation or with intensities below the background were not taken into account. Only spots, which passed these quality controls on 100% of arrays, were selected for further analysis. Data were analysed with GeneSpring GX V.13.0 (Agilent Technologies). ANOVA test (p value < 0.05) with Benjamini Hodgberg correction to check the False Discovery Rate (FDR) was used to determine the statistical significance in gene expression levels according to time (H0, H5, H20 and H45). Hierarchical clustering was performed with Pearson correlation metric and Ward’s linkage. K-means (K = 7) was performed with Pearson correlation metric. The Gene Ontology analysis was used to investigate the biological processes, molecular function or cellular localisation enriched in the transcript list showing a significant gene expression fluctuation according to time. The *p*-value was computed by standard hypergeometric distribution (*p*-value < 0.05). A GeneSpring Single Experiment Analysis (SEA) bio-informatics tool using WikiPathways database was used for computational analysis to identify potential curated canonical pathways which are enriched in the differentially expressed transcripts list. The significance of pathway enrichment was measured by standard hypergeometric distribution (*p*-value < 0.05).

## Additional Information

**How to cite this article**: Guillou, C. *et al.* Soluble alpha-enolase activates monocytes by CD14-dependent TLR4 signalling pathway and exhibits a dual function. *Sci. Rep.* **6**, 23796; doi: 10.1038/srep23796 (2016).

## Supplementary Material

Supplementary Information
