# Supplementary material for: Soluble alpha-enolase activates monocytes by CD14-dependent TLR4 signalling pathway and exhibits a dual function
Source: Sci Rep. 2016 Mar 30;6:23796. doi: 10.1038/srep23796 (PMC4824496; doi:10.1038/srep23796)
Supplement: Supplementary Information [file srep23796-s1.pdf]

Soluble alpha-enolase activates monocytes by CD14-dependent TLR4 signalling pathway and exhibits a dual function

Clément Guillou[1,*], Manuel Fréret[1,2,*], Emeline Fondard[1], Céline Derambure[1], Gilles Avenel[2], Marie-Laure Golinski[1,3], Mathieu Verdet[2], Olivier Boyer[1,4], Frédérique Caillot[1,3], Philippe Musette[1,3], Thierry Lequerré[1,2], Olivier Vittecoq[1,2]

[1]INSERM, U905 & Normandy University, Institute for Research and Innovation in Biomedicine (IRIB), Rouen, France
[2]Rouen University Hospital, Department of Rheumatology, Rouen, France
[3]Rouen University Hospital, Department of Dermatology, Rouen, France
[4]Rouen University Hospital, Department of Immunology, Rouen, France
*These authors contributed equally to this work

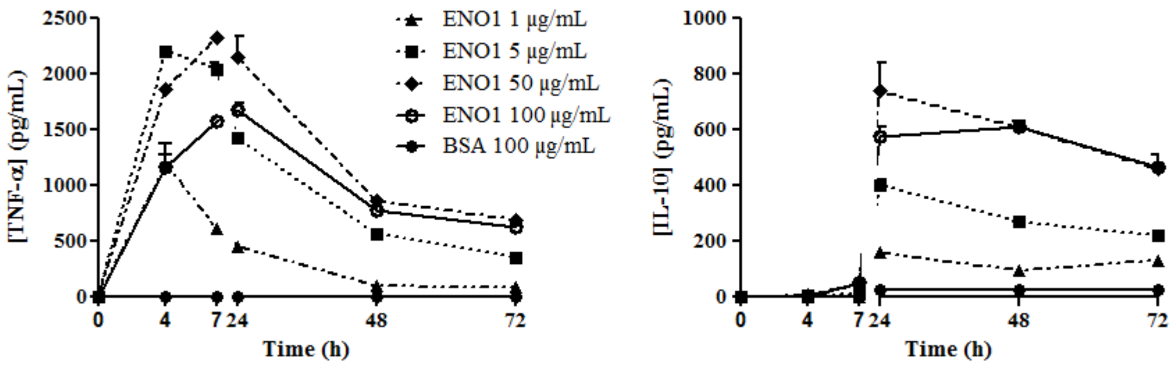

Sup. Figure 1. Dose-response study for production of TNF-α and IL-10 by PBMC stimulated with ENO1. 1.10^6 PBMC from healthy donors were incubated with different doses of ENO1 (1, 5, 50 or 100 µg/mL) or control BSA (100 µg/mL) and cultured for 72 hours. Supernatants were removed at different times (H0, H4, H7, H24, H48, and H72) and TNF-α and IL-10 levels were measured by ELISA. Data are expressed as mean ± SEM.

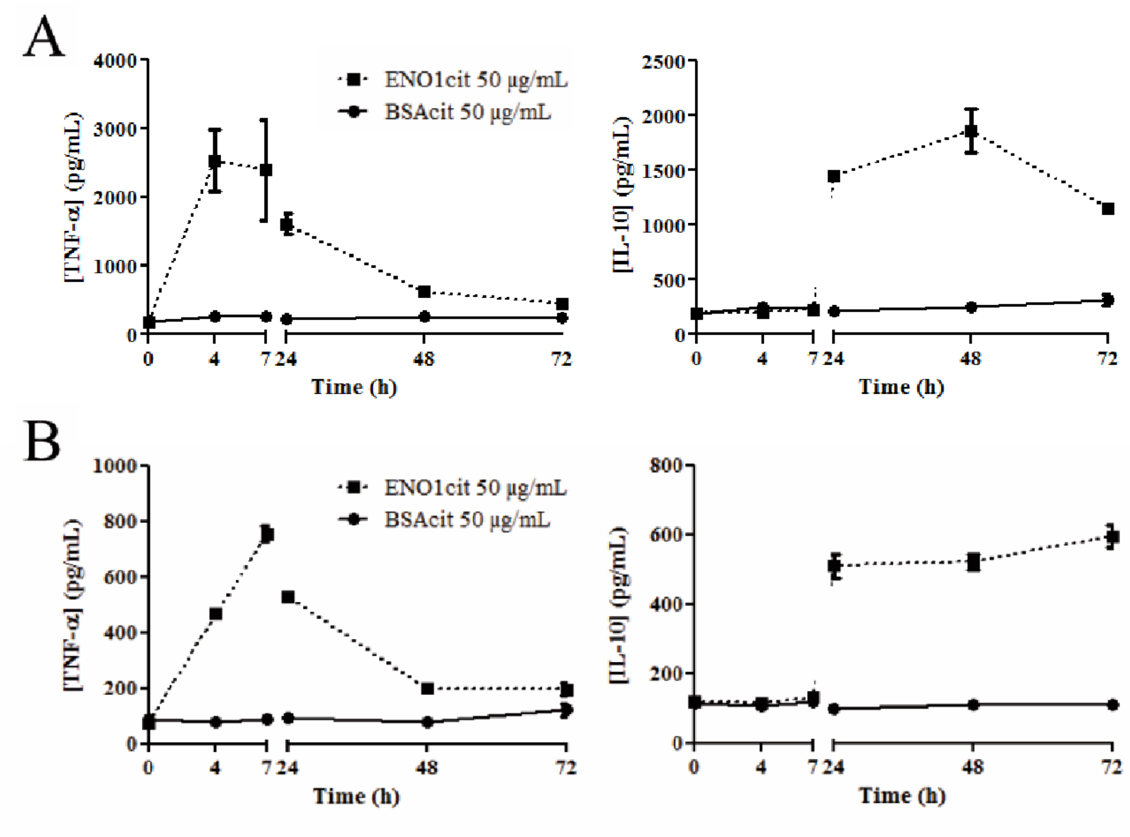

Sup. Figure 2. Citrullinated ENO1 induces an early production of TNF-α and a delayed IL-10 production in PBMC from healthy donors and RA patients. To investigate a potential pro- or anti-inflammatory effect of citrullinated ENO1, 1.10^6 PBMC from healthy donors (A) or RA patients (B) were incubated with citrullinated ENO1 (ENO1cit) (50 µg/mL) or citrullinated BSA (BSAcit) (50 µg/mL) and cultured for 72 hours. Supernatants were removed at different times (H0, H4, H7, H24, H48, and H72) and TNF-α and IL-10 levels were measured by ELISA. Data are expressed as mean ± SEM (n = 2).

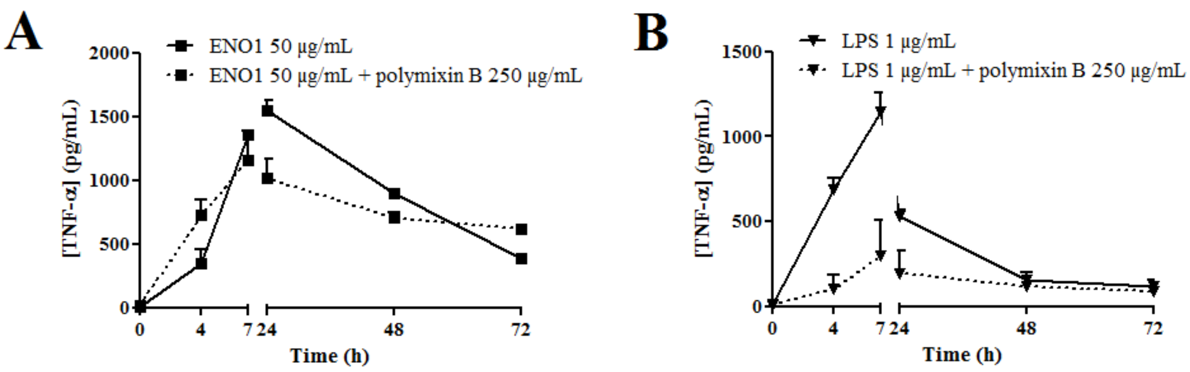

Sup. Figure 3. Polymixin B inhibits TNF-α production by LPS stimulated PBMC but no by ENO1 stimulated PBMC. To assess the hypothetical effect of residual LPS in ENO1 solution produced in *E.coli* (after endotoxin removal), 1.10^6 PBMC from healthy donors were stimulated with (A) ENO1 (50 µg/mL) or (B) LPS (1 µg/mL) with or without polymixin B (250 µg/mL), a specific LPS inhibitor. Supernatants were removed at different times (H0, H4, H7, H24, H48, and H72) and TNF-α level was measured by ELISA. Data are expressed as mean ± SEM.

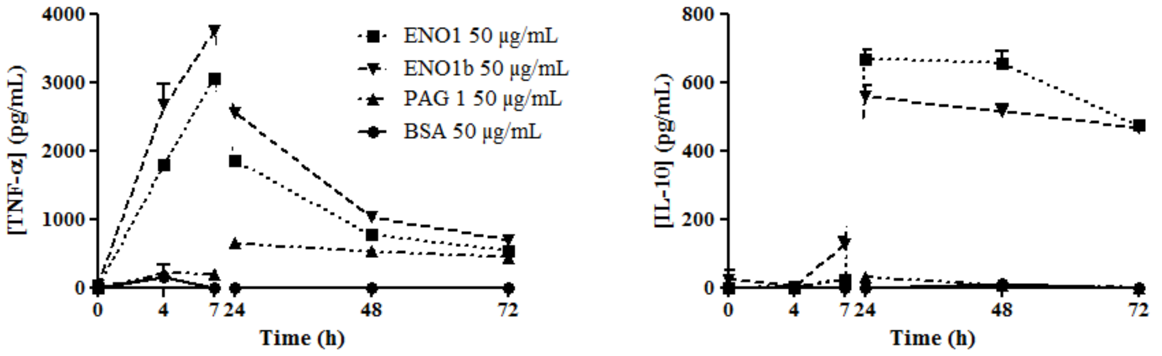

Sup. Figure 4. PBMC cytokines production induced by ENO1 is not due to residual LPS resulting in production in *E.coli*. To confirm absence of an hypothetical effect of residual LPS in ENO1 solution produced in *E.coli* (after endotoxin removal), 1.10^6 PBMC from healthy donors were stimulated with ENO1 (50 µg/mL), ENO1b (ENO1 produced with LPS-free baculovirus-insect cell expression system, 50 µg/mL), PAG 1 (produced in *E.coli*, 50 µg/mL) or control BSA (50 µg/mL). Supernatants were removed at different times (H0, H4, H7, H24, H48, and H72) and TNF-α and IL-10 levels were measured by ELISA. Data are expressed as mean ± SEM.

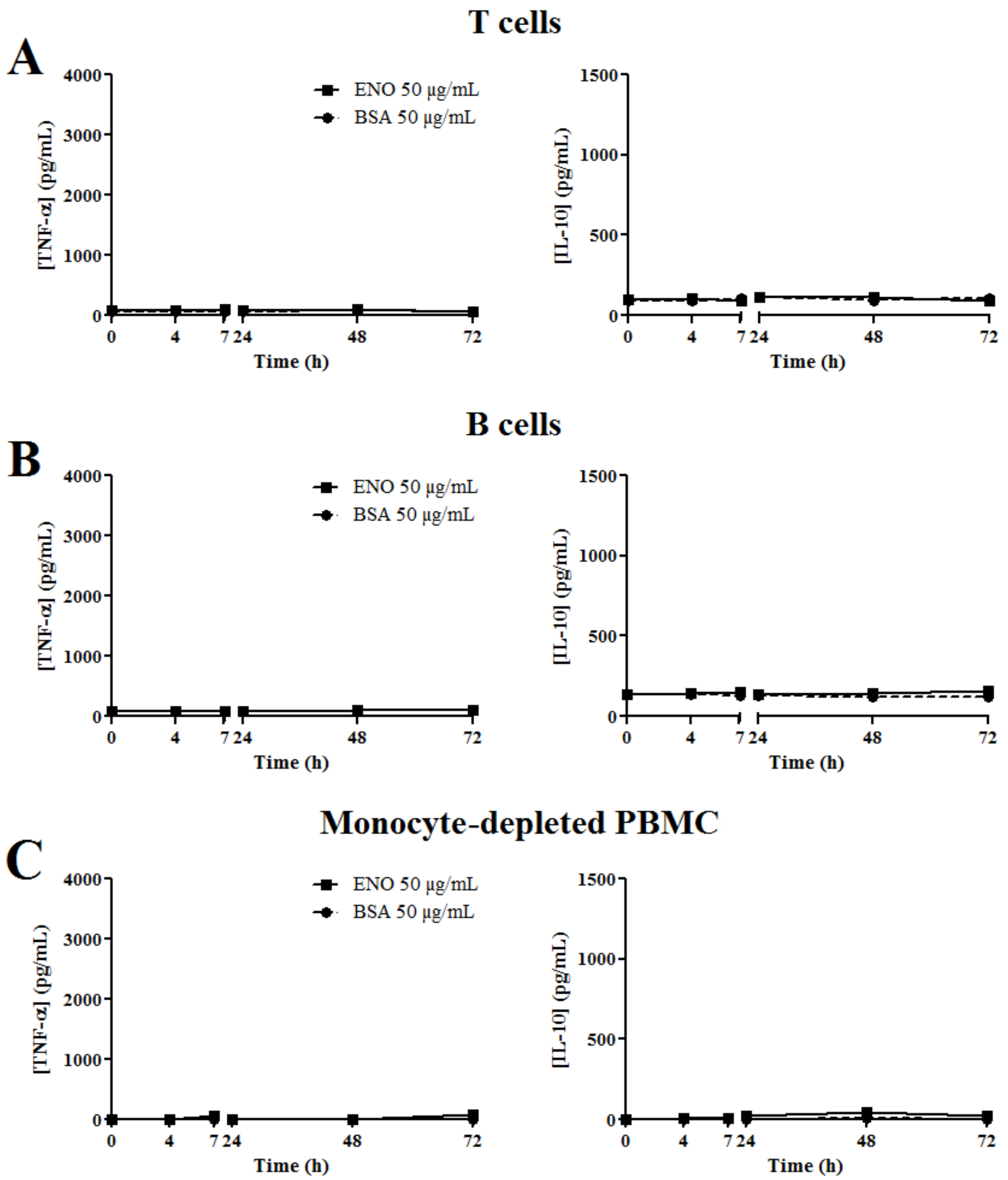

Sup. Figure 5. ENO1 stimulated T cells, B cells and monocytes-depleted PBMC do not produce TNF-α and IL-10. 1.10^{6} T cells (A), B cells (B) or monocyte-depleted PBMC (C) from healthy donors were cultured with ENO1 (50 µg/mL) or control BSA (50 µg/mL). Supernatants were removed at different times (H0, H4, H7, H24, H48, and H72) and TNF-α and IL-10 production was measured by ELISA. Data are expressed as mean ± SEM.

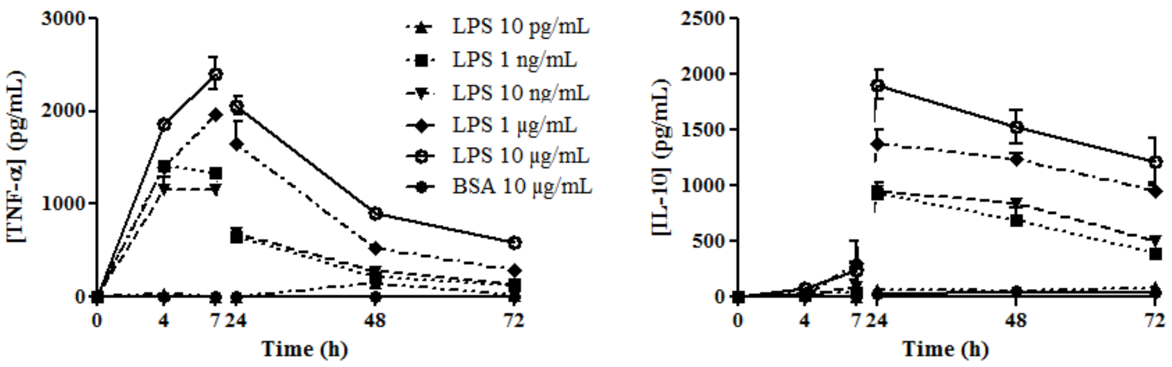

Sup. Figure 6. Dose-response study for production of TNF-α and IL-10 by PBMC stimulated with LPS. 1.10^6 PBMC from healthy donors were incubated with different doses of LPS (10 pg/mL, 1 ng/mL, 10 ng/mL, 1 µg/mL or 10 µg/mL) and cultured for 72 hours. Supernatants were removed at different times (H0, H4, H7, H24, H48, and H72) and TNF-α and IL-10 levels were measured by ELISA. Data are expressed as mean ± SEM.

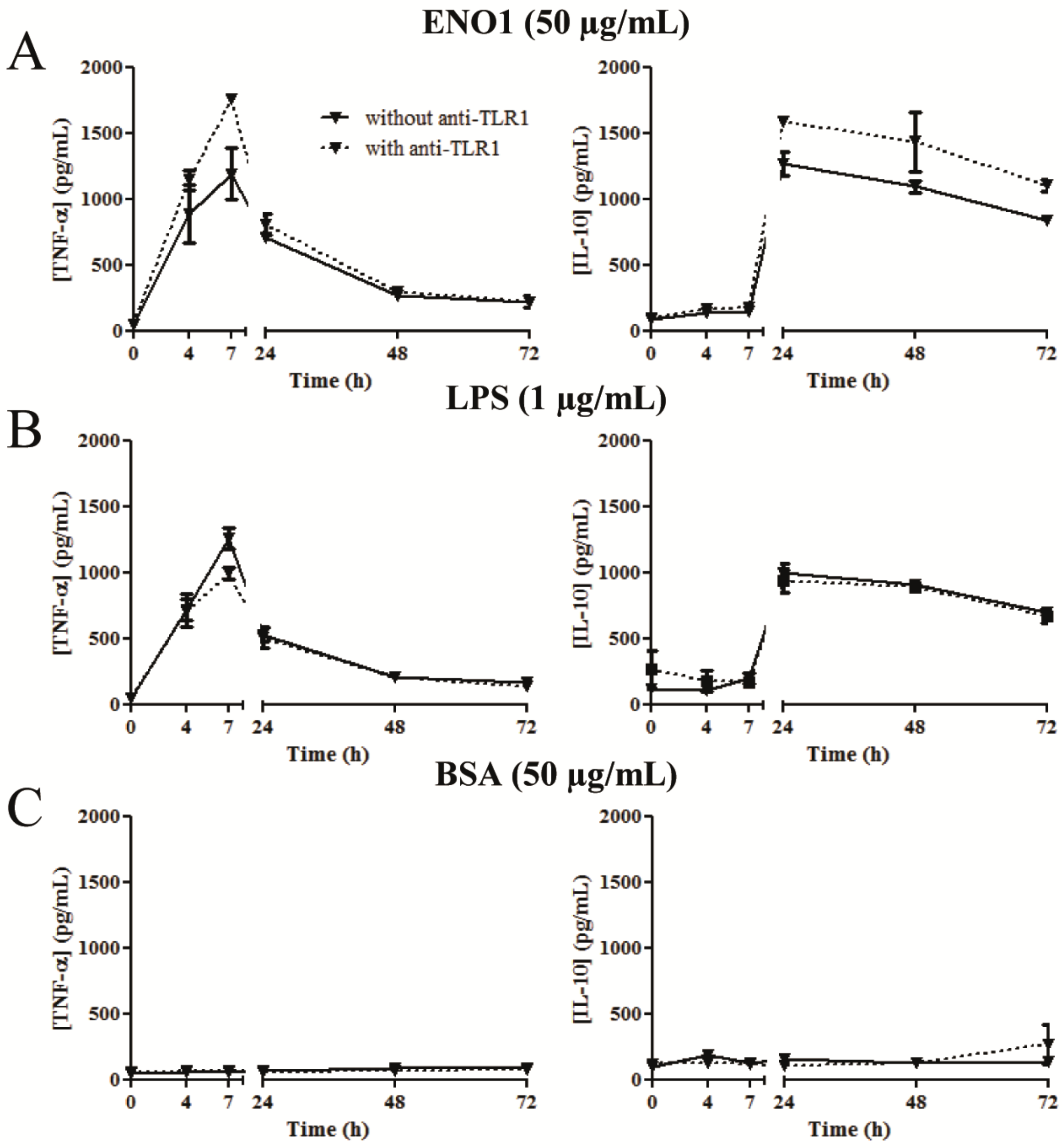

Sup. Figure 7. Blockade of TLR1 pathway does not modify the TNF-α and IL-10 production induced by ENO1. 1.10^6 PBMC from healthy donors were cultured with ENO1 (50 µg/mL) (A), LPS (1 µg/mL) (B) or BSA (50 µg/mL) (C) with (at 5 µg/ml) or without anti-TLR1 antibodies. Supernatants were removed at different times (H0, H4, H7, H24, H48, and H72) and cytokines (TNF-α and IL-10) production was measured by ELISA. Data are expressed as mean ± SEM (n = 2).

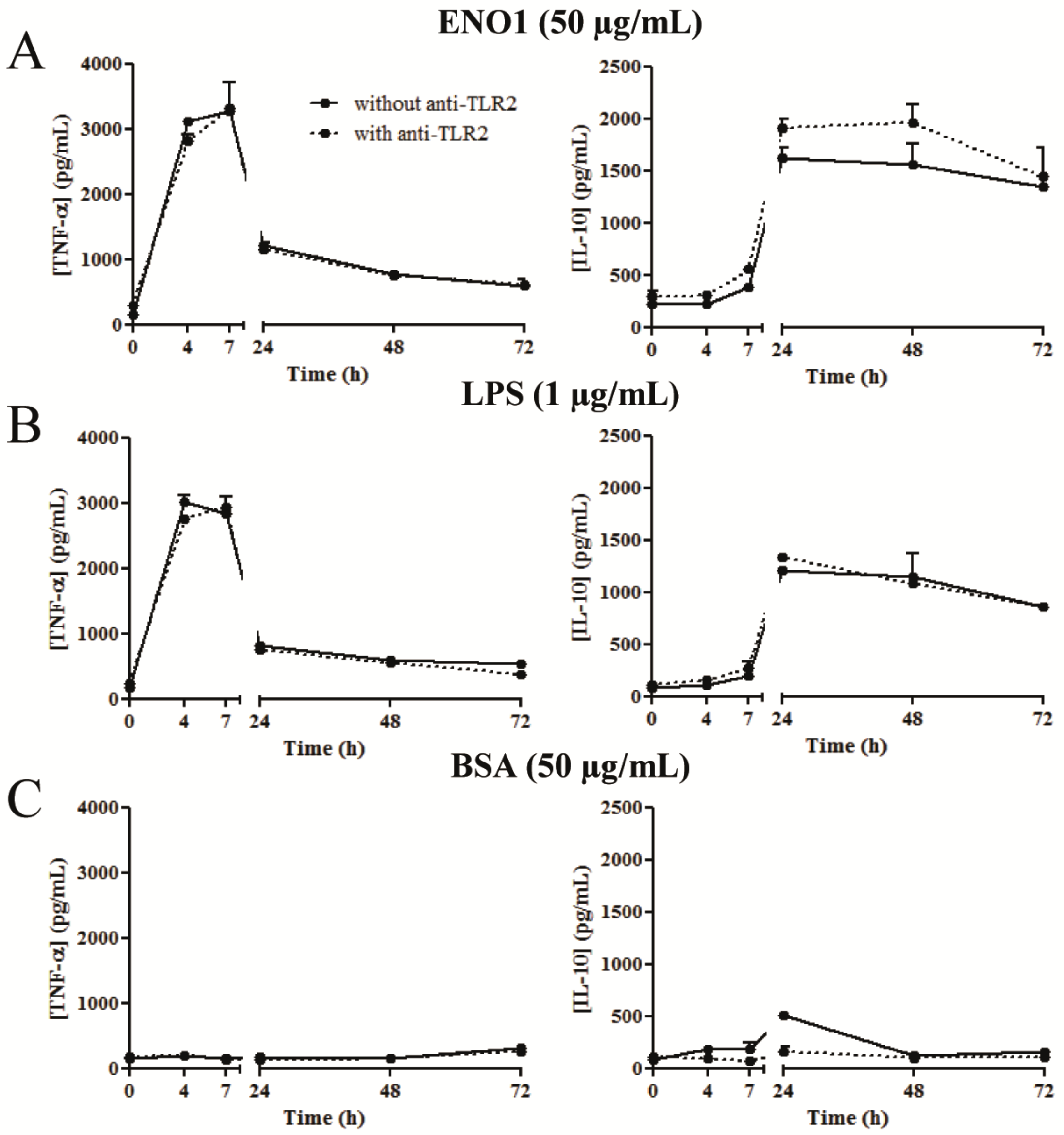

Sup. Figure 8. Blockade of TLR2 pathway does not modify the TNF-α and IL-10 production induced by ENO1. 1.10^6 PBMC from healthy donors were cultured with ENO1 (50 µg/mL) (A), LPS (1 µg/mL) (B) or BSA (50 µg/mL) (C) with (at 5 µg/ml) or without anti-TLR2 antibodies. Supernatants were removed at different times (H0, H4, H7, H24, H48, and H72) and cytokines (TNF-α and IL-10) production was measured by ELISA. Data are expressed as mean ± SEM (n = 2).

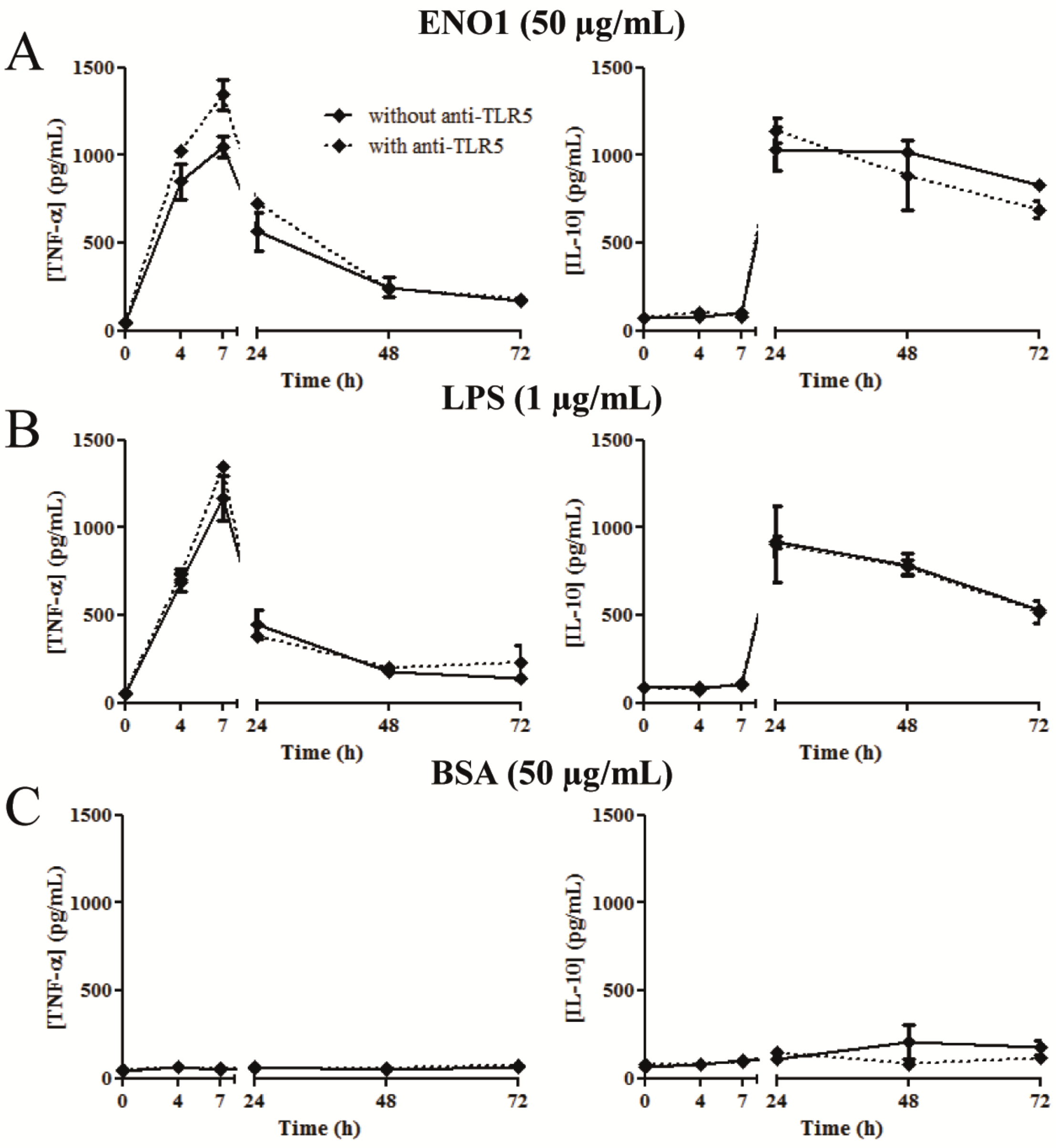

Sup. Figure 9. Blockade of TLR5 pathway does not modify the TNF-α and IL-10 production induced by ENO1. 1.10^6 PBMC from healthy donors were cultured with ENO1 (50 µg/mL) (A), LPS (1 µg/mL) (B) or BSA (50 µg/mL) (C) with (at 5 µg/ml) or without anti-TLR5 antibodies. Supernatants were removed at different times (H0, H4, H7, H24, H48, and H72) and cytokines (TNF-α and IL-10) production was measured by ELISA. Data are expressed as mean ± SEM (n = 2).

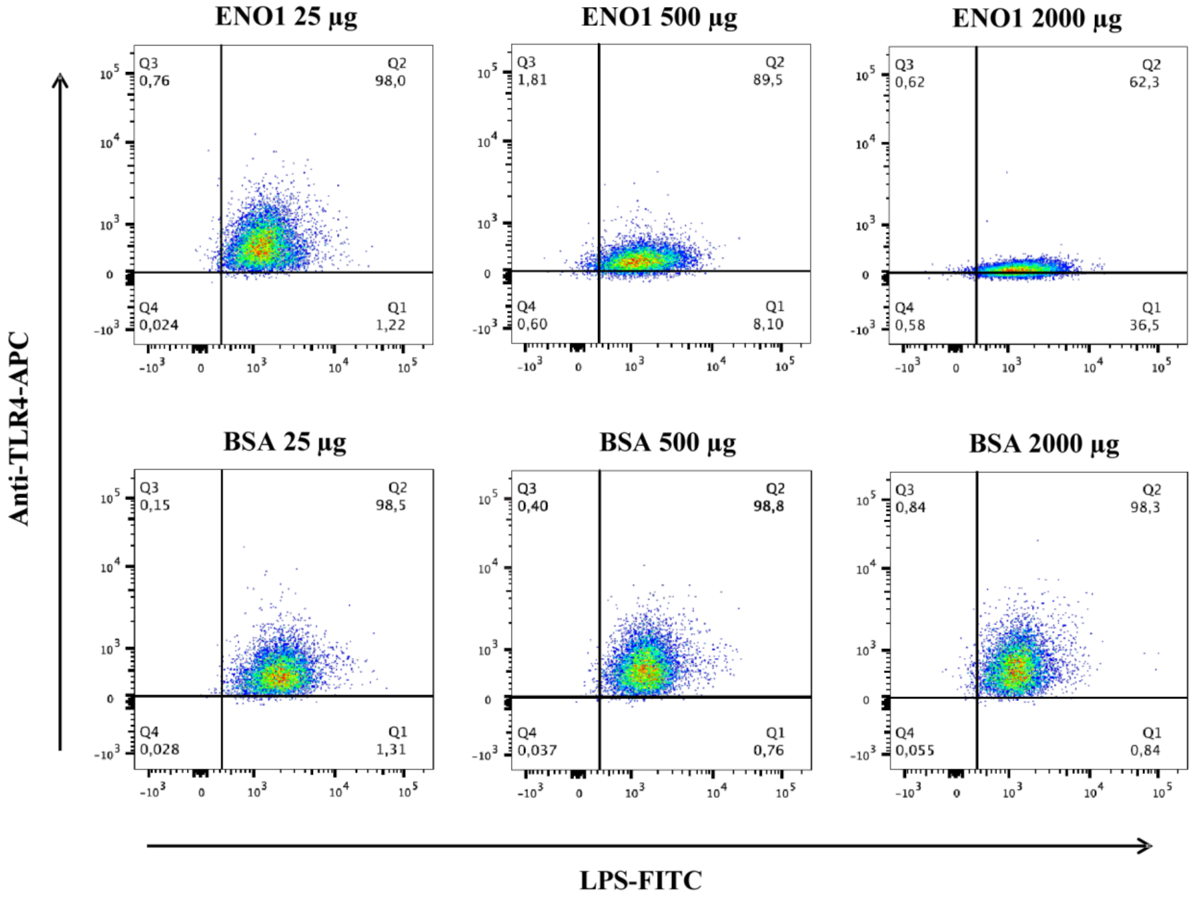

Sup. Figure 10. ENO1 binds to TLR4 but does not prevent the binding of LPS. 1.10^6 HEK-Blue hTLR4 cells were cultured with ENO1 (25 µg, 500 µg or 2 mg) for 20 minutes. FITC-labeled LPS (55 µg) and APC-labeled anti-TLR4 antibody were added for 20 minutes. Expression levels of FITC and APC on cells surface were determined by flow cytometry. Data are expressed as percentage of cells.

Sup. Table 1: List of 223 genes differently dysregulated in PBMCs stimulated with ENO1 compared to control BSA.

Gene	p (Corr)	Log FC (H5 vs H0)	Regulation	Log FC (H20 vs H0)	Regulation	Log FC (H45 vs H0)	Regulation
TREM1	0.00143593	-0.45533434	down	3.3546562	up	3.3884032	up
CNP	0.01624433	1.88207	up	0.90125203	up	0.49014032	up
IL1B	0.02721229	3.9511435	up	7.678227	up	6.461319	up
HERC6	0.02721229	3.9371443	up	2.3098898	up	1.7025051	up
ARAP3	0.02721229	-1.6712952	down	-0.18723828	down	1.0737756	up
ANKZF1	0.02721229	-0.7270257	down	-0.2249835	down	0.32215086	up
CXCL2	0.02721229	2.7633479	up	3.9431908	up	3.3564665	up
UBFD1	0.02721229	0.85809153	up	0.6355825	up	0.016682737	up
DNASE1L1	0.02721229	-1.8662013	down	-0.29480276	down	-0.21534543	down
FHOD1	0.02957191	-1.7403492	down	-0.34319595	down	0.046297092	up
RGMB	0.02957191	-0.17201434	down	-0.6313018	down	0.064362906	up
RAB11FIP4	0.02957191	-0.25966296	down	-0.00891305	down	0.5911951	up
ACTA2	0.02957191	1.3620137	up	0.6369272	up	0.8845734	up
OAS2	0.02957191	3.1647255	up	1.8221444	up	0.7000568	up
IFIT5	0.02957191	2.8443403	up	1.6464653	up	0.71543664	up
HERC5	0.02957191	4.077649	up	1.7460009	up	0.4499011	up
IRF7	0.02957191	2.5873568	up	1.8405253	up	1.0452942	up
LTBP3	0.02957191	-0.8050048	down	-0.4857471	down	0.30419096	up
BATF	0.02957191	2.6404572	up	1.552549	up	0.602088	up
SAP18	0.02957191	0.15704282	up	-0.04928427	down	-0.16246697	down
SULT1A2	0.02961564	-0.87628514	down	-0.46310356	down	-0.37910172	down
SLC26A6	0.02961564	-1.1487331	down	-0.39006197	down	0.29147974	up
HENMT1	0.02961564	-0.37938273	down	-0.20452309	down	0.07615573	up
DTX3L	0.02961564	2.5763857	up	1.4176179	up	0.51787204	up
LOC285074	0.02961564	-0.37044474	down	-0.14552148	down	0.39722058	up
SH3BP1	0.02961564	-0.7531193	down	-0.6097736	down	-0.09211234	down

PPP1R26	0.02961564	-1.4793859	down	-0.28289208	down	-0.75419074	down
EVA1B	0.02961564	-0.9596381	down	-0.02326306	down	-0.8481881	down
KIAA0125	0.02961564	-0.55897766	down	-0.35746908	down	0.48498568	up
SOCS1	0.02961564	1.8564287	up	2.3611672	up	2.0668237	up
SP110	0.02961564	2.221388	up	0.8102226	up	0.57383054	up
CXCL3	0.03216367	2.7799537	up	4.1639657	up	3.254077	up
FCAR	0.03216367	0.07995099	up	2.1529775	up	2.7386913	up
SP100	0.03216367	1.5547333	up	0.7667993	up	0.41499642	up
STMN3	0.03216367	-0.96635365	down	-0.43274713	down	0.1390817	up
P4HTM	0.03223528	-0.5941391	down	-0.062177315	down	0.35054216	up
FXYD6	0.03347715	-0.6050727	down	0.5440297	up	-1.3821362	down
SERPINB2	0.03347715	4.4634814	up	6.7400165	up	4.700161	up
TRIP12	0.03347715	0.29560757	up	0.02803804	up	0.1222471	up
MMS19	0.03347715	-0.41863465	down	-0.27180764	down	0.1091725	up
HDAC7	0.03347715	-1.0749317	down	-0.3166583	down	0.25476745	up
LRFN4	0.03347715	-0.9957079	down	0.06023832	up	-0.63982373	down
SAMD9	0.03347715	2.2437418	up	0.8170131	up	0.32005534	up
HIP1R	0.03347715	-0.63205403	down	-0.044148788	down	0.6317101	up
EIF2AK2	0.03347715	3.2713416	up	1.9491237	up	0.6280526	up
SLC25A29	0.03347715	-1.349244	down	-0.4066362	down	-0.14098443	down
SEMA6B	0.03347715	-2.1732628	down	0.062537394	up	-0.027618995	down
SAMD9	0.03347715	2.2554362	up	0.74211246	up	0.26097712	up
RTKN2	0.03347715	0.68665475	up	0.25625834	up	0.3650432	up
MX1	0.03347715	3.7743394	up	2.7784548	up	0.8971815	up
GDPD5	0.03347715	-0.8798832	down	-0.5071185	down	0.08747161	up
RNPEPL1	0.03347715	-0.95807624	down	-0.36051658	down	-0.10479986	down
HIP1R	0.03347715	-0.79036444	down	0.14403088	up	0.9204759	up
IFIT5	0.03347715	2.6029313	up	1.6948265	up	0.6481905	up
LINS	0.03347715	1.0076799	up	0.4381505	up	0.42563727	up
LOC102724364	0.03347715	0.004854133	up	0.12473193	up	-1.0596132	down
ARMCX1	0.03347715	0.35678196	up	-0.33336118	down	-2.0410163	down

GYS1	0.03347715	-0.72118574	down	-0.2649809	down	0.15248276	up
XYLT1	0.03347715	-0.69423264	down	-1.3096198	down	-0.5722688	down
TAF4B	0.03369069	2.2829986	up	0.94330025	up	0.8919994	up
PIH1D1	0.03486062	-0.96135956	down	-0.38228726	down	-0.26547277	down
TRIM24	0.03554605	-0.20674825	down	-0.58906716	down	-0.6404988	down
CORO2A	0.03565979	-1.4465936	down	-0.55626774	down	0.43222865	up
STMN3	0.03565979	-1.0564137	down	-0.5778122	down	0.11638575	up
C3orf38	0.03565979	0.70243543	up	0.059360843	up	-0.11141681	down
PDLIM2	0.03565979	-1.0046169	down	-1.0103873	down	-0.47551474	down
OASL	0.03565979	2.3537989	up	1.7626249	up	1.3253118	up
PPP2R5B	0.03565979	-0.703228	down	-0.19903888	down	0.10338112	up
NSUN5	0.03677070	-0.3523185	down	-0.20406628	down	0.22585487	up
ZNF433	0.03879803	-0.6015973	down	0.43937364	up	0.3552842	up
PRELID1	0.03879803	-0.23562305	down	0.24960046	up	0.19561231	up
PTPN6	0.03879803	-1.112166	down	-0.31929305	down	0.032994542	up
WDR37	0.03879803	-0.74295837	down	-0.5158681	down	-0.2485113	down
C18orf25	0.03879803	1.5700232	up	0.70860654	up	0.3009009	up
DNAJA1	0.03889201	1.304552	up	0.3810618	up	-0.015176508	down
ZBTB40	0.03898457	-0.38555464	down	-0.27436486	down	0.20668155	up
HDAC7	0.03898457	-1.2087263	down	-0.3629125	down	0.3235458	up
DPYSL2	0.03898457	-1.2579778	down	-0.8110504	down	-1.670514	down
PKD1	0.03898457	-0.7439745	down	-0.28514123	down	0.31655264	up
MZF1	0.03925520	-0.6862809	down	-0.30311614	down	0.09523303	up
IFIH1	0.03959255	2.9185045	up	1.1428329	up	0.108953476	up
IFT172	0.03959255	-0.7875681	down	-0.48014608	down	-0.31194502	down
RIPK2	0.03959255	2.0718334	up	1.7704077	up	0.494586	up
FUT8	0.03959255	0.08301654	up	-0.32330826	down	0.059242513	up
LINC00341	0.03959255	-0.8663151	down	-0.75651044	down	-0.3647085	down
PAIP1	0.04047068	0.3600874	up	0.10178629	up	-0.4571514	down
LOC100132057	0.04047068	-1.204365	down	-1.1660193	down	-0.24738295	down
KLHDC8B	0.04047068	-0.5765729	down	-0.149063	down	-1.6347634	down

ENKD1	0.04047068	-0.9922208	down	-0.2476297	down	0.07499572	up
CAB39L	0.04047068	0.70884657	up	-0.039463054	down	-0.35634875	down
USP47	0.04047068	-0.17315465	down	-0.36676267	down	0.22950344	up
HERC1	0.04047068	-0.61629206	down	0.03141718	up	0.28270113	up
UPF3A	0.04047068	-0.62302846	down	-0.5499261	down	-0.13542841	down
TRAPPC6B	0.04047068	-0.1885281	down	-0.52871513	down	-0.15958802	down
CXCL5	0.04047068	1.5604714	up	4.647899	up	5.119076	up
DTX3L	0.04047068	2.7398307	up	1.5050398	up	0.925061	up
IER2	0.04056386	-0.1518835	down	-0.4116086	down	0.45367494	up
SP100	0.04094664	1.6633266	up	0.4516963	up	0.37408674	up
WDR73	0.04094664	-0.53893393	down	-0.09606717	down	0.16737197	up
FLI1	0.04094664	0.72549886	up	-0.5004781	down	-0.2828044	down
GPC2	0.04094664	-1.5743631	down	0.11780705	up	1.3819634	up
WDR73	0.04094664	-0.46531573	down	-0.30157208	down	0.1921376	up
ANKRD54	0.04094664	-0.2844129	down	-0.4934585	down	0.20246853	up
SH3BP5L	0.04094664	-0.49987313	down	-0.38429806	down	-0.1568604	down
USP3	0.04094664	-0.3893051	down	0.15102477	up	0.3074039	up
NFATC3	0.04094664	-0.59564435	down	-0.5056702	down	-0.16985923	down
KCNQ1	0.04094664	-0.96499604	down	-1.513374	down	-1.2256842	down
BIN2	0.04094664	-0.59089243	down	-0.781765	down	-0.32749557	down
LARP4	0.04094664	0.69166183	up	0.19837952	up	-0.015357628	down
MAPKBP1	0.04094664	-0.9369836	down	-0.21234326	down	0.21564072	up
DPYSL2	0.04094664	-1.0743262	down	-0.7212766	down	-1.5107542	down
MCOLN2	0.04094664	1.8208126	up	1.6050835	up	-0.3861748	down
ULK1	0.04094664	-0.9854501	down	-0.01888599	down	0.45760372	up
MGAT4A	0.04094664	-0.021560976	down	-0.3607912	down	-1.1406422	down
MUM1	0.04094664	-1.1224617	down	-0.30752373	down	0.2701505	up
HSPH1	0.04094664	0.65367544	up	0.11467084	up	-0.36267912	down
LOC100132057	0.04094664	-1.3810353	down	-1.2447712	down	-0.22252376	down
NDRG2	0.04094664	-2.0042305	down	-0.81246334	down	0.0681887	up
SLC16A5	0.04094664	-2.7091074	down	-1.219945	down	-1.2359844	down

TM9SF3	0.04094664	0.98229885	up	0.09324054	up	-0.2888066	down
	0.04094664	-1.0187984	down	-0.68638754	down	0.1727277	up
TIPRL	0.04094664	0.37386575	up	0.35580468	up	-0.0377112	down
SLC39A13	0.04094664	-1.1181806	down	-0.08066432	down	0.4747053	up
IL3RA	0.04094664	-0.44443047	down	1.4486743	up	2.5156386	up
PITPNM2	0.04094664	-1.8303951	down	-1.5576435	down	-0.4021765	down
KDELR2	0.04094664	0.3947784	up	-0.060978442	down	-0.44554082	down
DND1	0.04149704	-0.6081155	down	-0.23261362	down	0.37998036	up
DEFA3	0.04149704	0.039506003	up	-0.51154613	down	-5.34037	down
CD274	0.04162654	2.4875858	up	2.4041188	up	1.0412151	up
ACADVL	0.04162654	-1.0882334	down	-0.29974046	down	0.20515132	up
ARHGEF4	0.04162654	-0.57717973	down	-1.7438339	down	-0.15808071	down
SAMHD1	0.04162654	1.2568212	up	-0.6554011	down	-1.2536436	down
BAZ1A	0.04171938	1.1493431	up	0.482584	up	0.08689789	up
TRABD	0.04171938	-0.7555304	down	-0.018592855	down	0.6788237	up
HPS4	0.04171938	-0.4906797	down	-0.13629588	down	-0.062473547	down
CCL3L3	0.04171938	2.745622	up	3.293374	up	2.690502	up
HIP1	0.04171938	-1.0969719	down	-0.2616681	down	0.23847704	up
PIGQ	0.04171938	-0.9285795	down	-0.28899574	down	-0.11281621	down
BIN3	0.04171938	-0.5764497	down	-0.11267415	down	0.15141706	up
GNA12	0.04171938	-0.84078854	down	0.15398562	up	0.30925205	up
HSD17B4	0.04171938	-0.56935924	down	-0.36470863	down	-1.468051	down
ZKSCAN3	0.04171938	-0.91421705	down	-1.3467356	down	-0.30368242	down
MRPL39	0.04171938	0.4024407	up	-0.12662633	down	-0.23087496	down
C16orf74	0.04171938	-1.0952693	down	-1.1907841	down	-0.16481315	down
MTERF	0.04171938	0.34369484	up	0.02585418	up	0.17426772	up
CD47	0.04185472	0.76394016	up	-0.2564262	down	0.11090212	up
IFI6	0.04260640	3.7601547	up	2.925811	up	0.11501113	up
STARD9	0.04260640	-0.77342874	down	-0.8045152	down	0.3129044	up
SEC14L1	0.04306389	-0.47637525	down	0.21898776	up	-0.28308967	down
ME1	0.04306389	-0.33457384	down	-1.3094522	down	-3.3739986	down

TFE3	0.04360148	-0.5497628	down	0.41146243	up	0.12911157	up
PMEPA1	0.04360148	0.50249743	up	1.0206403	up	1.9013506	up
CCS	0.04360148	-0.90388936	down	-0.32065895	down	-0.013087937	down
SNRPB2	0.04360148	0.44650745	up	0.107287966	up	-0.23893745	down
SLC35E2	0.04360148	-0.77599716	down	-0.44656682	down	0.121189445	up
BRD7P3	0.04360148	0.502057	up	0.03723172	up	-0.028463775	down
RNH1	0.04360148	-0.76384807	down	-0.31183687	down	-0.83633775	down
TMEM8B	0.04360148	-1.1847609	down	-0.03902184	down	0.37666416	up
RASSF1	0.04360148	-0.8200881	down	-0.7930155	down	-0.5099596	down
BMPR2	0.04360148	0.6735944	up	-0.61333394	down	-0.50719595	down
ARRB2	0.04360148	-1.1607618	down	-0.41954622	down	0.15347958	up
STK25	0.04360148	-0.6753769	down	-0.09984651	down	0.18886949	up
LEPREL1	0.04360148	0.05507465	up	-0.046279687	down	-2.127578	down
NCK1	0.04360148	0.089600526	up	-0.15428992	down	-0.75705177	down
	0.04360148	-0.768753	down	-0.32570255	down	-0.11007049	down
OAS3	0.04360148	4.145535	up	2.8464386	up	0.5505845	up
B3GNT1	0.04360148	-1.3227129	down	-0.3475025	down	0.013157544	up
TCF25	0.04360148	-0.5133336	down	-0.42090476	down	-0.21282674	down
PEX3	0.04360148	0.44046125	up	-0.037348885	down	-0.21600266	down
FKBP1A	0.04360148	-0.5066989	down	0.123146035	up	-0.11110779	down
STK25	0.04369362	-0.720679	down	-0.08482495	down	0.050629884	up
CCL3	0.04425991	3.702957	up	4.6452484	up	3.379403	up
HMGN1	0.04432157	-0.3973199	down	0.22166336	up	0.19484697	up
BCL10	0.04432157	0.40901574	up	-0.23793085	down	-0.55884033	down
STOM	0.04441009	1.1411968	up	0.1573814	up	-0.10847408	down
OSGIN2	0.04441009	0.9361191	up	1.0723289	up	-0.02077832	down
NFKBIZ	0.04441009	1.2209857	up	0.9365018	up	1.0268668	up
TRIM22	0.04467685	2.9447343	up	1.6871914	up	0.847128	up
IL3RA	0.04543779	-0.7584133	down	1.5026792	up	2.5442226	up
	0.04562725	2.3023856	up	0.71731836	up	0.13691504	up
FUNDC1	0.04586121	0.5258701	up	0.038215436	up	-0.30227044	down

BACE1	0.04664252	-0.819563	down	-0.81954765	down	-2.7396686	down
TMUB2	0.04664252	-0.41432288	down	0.040623013	up	0.15970893	up
	0.04901225	-0.6086789	down	-0.73561215	down	-0.07093814	down
GLIS3	0.04901225	0.7102484	up	1.9836521	up	3.0786371	up
SAR1B	0.04967081	0.44899043	up	0.26688343	up	-0.7483134	down
ZFYVE26	0.04967081	0.74382645	up	-0.11025231	down	-0.36943603	down
ZMIZ2	0.04967081	-1.0975624	down	-0.13498561	down	0.23207569	up
C18orf25	0.04967081	1.4615542	up	0.61479753	up	0.46532497	up
IL1RN	0.04967081	3.306846	up	4.152791	up	3.5500777	up
MTA1	0.04967081	-0.5945975	down	-0.36207008	down	0.2767041	up
SEC22B	0.04967081	-0.18477385	down	0.13116743	up	-1.0062243	down
ZNF853	0.04967081	-0.5761639	down	-0.053330064	down	0.8294382	up
FECH	0.04967081	0.12711215	up	-0.44342765	down	-1.2386206	down
NIPSNAP1	0.04967081	-0.46140972	down	-0.18091464	down	0.39589134	up
CCL3L3	0.04967081	3.3978405	up	4.3306785	up	3.1164014	up
DALRD3	0.04967081	-0.87449616	down	-0.354764	down	0.23140359	up
ARNTL	0.04967081	-0.49137786	down	-0.3226172	down	0.19147928	up
SKIV2L	0.04967081	-0.7747368	down	-0.4335321	down	0.055167288	up
SAMD9	0.04967081	2.4332402	up	0.83604175	up	0.5491468	up
BTG2	0.04967081	-0.29564825	down	-0.020069405	down	0.84014827	up
C11orf21	0.04967081	-2.1156304	down	-1.6078621	down	-0.91792136	down
TRIB2	0.04967081	1.3510771	up	-0.29101765	down	0.6029823	up
AZI2	0.04967081	0.9143962	up	0.0748869	up	-0.22570175	down
TJP2	0.04967081	0.9322226	up	0.48574767	up	-0.73905444	down
	0.04967081	-0.66981965	down	2.88E-04	up	-0.91259104	down
	0.04967081	-1.2936825	down	-0.28206578	down	0.74589187	up
PIM2	0.04967081	1.1548704	up	0.9279639	up	0.8897343	up
PPP1R13B	0.04967081	-0.324729	down	-0.1700025	down	1.0722526	up
IFIT2	0.04967081	3.9225407	up	2.1824381	up	1.1813968	up
STOM	0.04967081	1.3465062	up	0.37389138	up	0.23148935	up
WDR34	0.04967081	-0.77209026	down	-0.0783632	down	0.50322014	up

CMTM8	0.04967081	1.2198353	up	1.0032454	up	1.5225512	up
ATP2A3	0.04967081	-0.6681464	down	-0.23012412	down	0.7623145	up
TRIM22	0.04967081	2.8151429	up	1.6413246	up	0.76597756	up
IFFO1	0.04967081	-1.8720335	down	-0.7374859	down	-0.61568505	down
NACC2	0.04967081	-1.4842621	down	0.046779346	up	-0.42652833	down
KDSR	0.04967081	1.4697976	up	-0.1590641	down	-0.22224472	down
AP3S1	0.04967081	-0.21836154	down	-0.4999113	down	-0.63222665	down
SNPH	0.04967081	-1.5822382	down	-0.5630496	down	0.21938898	up
HTRA2	0.04967081	-0.6588272	down	-0.04018946	down	0.10361896	up
UBXN6	0.04967081	-0.57521826	down	-0.31563976	down	-0.39334738	down
ZNF234	0.04974354	-0.4473426	down	-0.36404827	down	0.08456839	up

Sup. Table 2: Related genes with biological processes identified by the gene ontology (GO) enrichment analysis.

Biological processes (GO)	Number of related genes	Genes
Innate immune response	15	CCL3, EIF2AK2, HERCC5, IFI6, IFIH1, IFIT2, IFIT5, IRF7, MX1, OAS2, OAS3, OASL, RIPK2, SOCS1, SP100
Inflammatory response	8	CCL3, CCL3L3, CXCL2, CXCL3, IL1B, ILARN, NFKBIZ, RIPK2
Leukocyte migration	5	CCL3L3, CCL3, IL1B, CXCL3, CXCL2
Response to cytokines	14	CCL3, EIF2AK2, HERCC5, IFI6, IFIT2, IL1B, IL1RN, IRF7, MX1, OAS2, OAS3, OASL, SOCS1, SP100
LPS signaling pathway	3	RIPK2, CCL3, IL1B
Response to IFN-I	9	IFI6, IFIT2, IRF7, Mx1, OAS2, OAS3, OASL, SOCS1, SP100
